# Multiple myeloma with spinal involvement: Renal dysfunction and albuminuria are associated with progressive sarcopenia in a longitudinal CT morphometric study

**DOI:** 10.1016/j.bas.2026.106153

**Published:** 2026-06-30

**Authors:** Julian Kylies, Dominik Kylies, Katja Weisel, Ulrich O. Wenzel, Tobias B. Huber, Lennart Viezens, Leon-Gordian Leonhardt

**Affiliations:** a Department of Trauma and Orthopedic Surgery, University Medical Center Hamburg-Eppendorf, Hamburg, Germany; b III. Department of Medicine, University Medical Center Hamburg-Eppendorf, Hamburg, Germany; c Hamburg Center for Kidney Health (HCKH), University Medical Center Hamburg-Eppendorf, Hamburg, Germany; d Department of Oncology, Hematology and Bone Marrow Transplantation with Section Pneumology, Hubertus Wald University Cancer Center Hamburg (UCCH), University Medical Center Hamburg-Eppendorf, Hamburg, Germany

**Keywords:** Multiple myeloma, Kidney dysfunction, Sarcopenia, Computed tomography morphometry, Body composition, Retrospective study

## Abstract

**Background:**

Kidney dysfunction is a frequent complication of multiple myeloma (MM). However, its associations with sarcopenia and visceral adipose tissue loss, emerging prognostic markers of frailty, remain insufficiently characterized in MM. Computed tomography (CT)-based morphometry provides an objective and reproducible assessment of body composition. Albuminuria, an established marker of kidney damage, is often underrecognized outside nephrology despite its potential relevance for disease burden and physical decline.

**Methods:**

We retrospectively analyzed 86 MM patients (2009–2024) with three sequential whole-body CT scans. CT morphometry included skeletal muscle index (SMI), paraspinal muscle index (PSMI), psoas muscle index (PMI), skeletal muscle density (SMD), and visceral adipose tissue (VAT). Kidney function (serum creatinine, eGFR, albuminuria) and serum albumin were assessed in relation to longitudinal changes in body composition and clinical outcomes, including Eastern Cooperative Oncology Group (ECOG) performance status, pain severity (VAS), and World Health Organization (WHO) analgesic use.

**Results:**

Both impaired baseline kidney function (eGFR <60 mL/min) and progressive eGFR decline (≥25 %) were associated with significantly accelerated loss of skeletal and visceral muscle parameters. Notably, patients with stable but impaired baseline kidney function also exhibited greater muscle and fat loss compared with those with preserved and stable renal function. Albuminuria >30 mg/24 h was also associated with adverse CT morphometry, irrespective of eGFR. In addition, baseline hypoalbuminemia (<3.5 g/dL) and longitudinal serum albumin declines ≥10 % correlated with unfavorable CT-morphometric trajectories. Functionally, progressive kidney dysfunction was associated with worsening ECOG performance status.

**Conclusions:**

Kidney dysfunction was independently associated with muscle and adipose tissue loss in MM patients. In addition, albuminuria represents an additional, underrecognized marker of progressive sarcopenia, even independent of eGFR. These findings underscore the importance of integrating renal parameters and sequential imaging-based assessments for risk stratification and supportive care planning in patients with MM.

## Highlights

• Kidney dysfunction is strongly associated with accelerated muscle and visceral adipose tissue loss in multiple myeloma patients.
• Both baseline renal impairment (eGFR <60 mL/min) and progressive decline (≥25 %) predict adverse CT-based body composition changes.
• Albuminuria (>30 mg/24 h) identifies patients at risk for sarcopenia independently of eGFR.
• Hypoalbuminemia and longitudinal albumin decline (≥10 %) correlate with unfavorable morphometric trajectories.
• Integration of renal biomarkers and CT morphometry may improve risk stratification and supportive care in MM.

## 1 Introduction

Kidney dysfunction is a hallmark feature of multiple myeloma (MM), a hematologic malignancy predominantly affecting the elderly (Turesson et al., 2010; Myeloma - Cancer Stat Facts). Despite advances in care, kidney dysfunction in MM is still associated with morbidity and reduced overall survival (Dimopoulos et al., 2016, 2017; Augustson et al., 2005). The pathophysiology of kidney dysfunction in MM is complex and includes cast nephropathy and monoclonal immunoglobulin depositions, among others (Leung et al., 2021; Dimopoulos et al., 2008). Furthermore, as the frequency of chronic kidney disease (CKD) increases with age, the prevalence of CKD in MM patients is high (Stevens and Levey, 2005; Liu et al., 2021). These factors place patients, who are often elderly, at particularly high risk for progressive clinical decline and frailty.

Frailty is a syndrome characterized by progressive sarcopenia and increased vulnerability (Cederholm and Bosaeus, 2024; Kim and Rockwood, 2024). It is associated with morbidity and mortality across a broad spectrum of medical fields, including oncology and CKD (Goede, 2023; Kennard et al., 2023), making it an emerging target for intervention. However, traditional frailty-assessments are time consuming (Kim and Rockwood, 2024), limiting their broader application. Sarcopenia, a frequent manifestation of frailty, has also been linked to increased morbidity and mortality (Brown et al., 2016; Allison et al., 1999). It can quickly and objectively be assessed utilizing CT-morphometrics therefore serving as a promising assessment tool (Chowdhury et al., 2018).

Recent studies showed that kidney dysfunction may directly accelerate and promote muscle loss through molecular mechanisms and inter-organ crosstalk, including inflammation, metabolic dysregulation, and protein-energy wasting (Kim and Rockwood, 2024; Puelles and Huber, 2022; Solagna et al., 2021). However, their relationships remain poorly understood. A deeper understanding could aid individualized treatment and risk stratification, especially since MM patients are at risk for both MM-induced kidney injury and pre-existing CKD. Furthermore, besides reductions in glomerular filtration rate, the role of albuminuria, a key kidney parameter in nephrology, yet often neglected in other disciplines, remains underexplored as well.

This study therefore aims to bridge this gap in knowledge by investigating the associations between kidney dysfunction and CT-morphometry parameters MM patients. To achieve this goal, we here analyzed a cohort of MM patients who underwent three sequential whole-body CT scans over the course of their disease to determine whether impaired kidney function and albuminuria contribute to an accelerated decline in muscle and adipose tissue. Furthermore, the associations between kidney dysfunction and functional status, pain levels, and analgesic consumption were assessed.

## 2 Materials and methods

### 2.1 Study design and outcome parameters

The study was approved by the local medical ethics board (ID: 2025-300576-WF) and conducted in accordance with the Declaration of Helsinki. Due to the retrospective and anonymized study design, informed consent was waived.

**Fig. 1:**
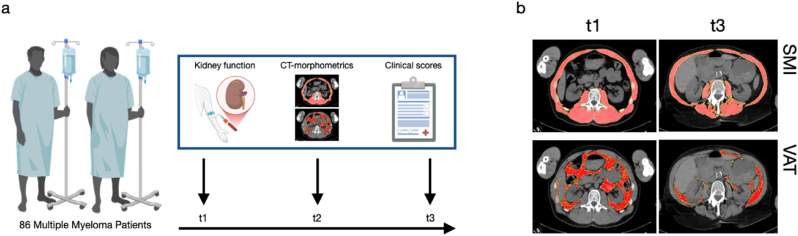
**| Study design and exemplary depiction of CT morphometry. a.** Retrospective study design. A total of 86 patients with multiple myeloma who underwent three whole-body CT scans at our university medical center between 2009 and 2024 were included. At each imaging time point (t1–t3), laboratory parameters including kidney function, as well as CT morphometry and clinical scores (ECOG performance status, VAS, and WHO pain medication), were assessed. **b.** Exemplary depiction of CT morphometry. Shown are axial CT slices at the level of the third lumbar vertebra (L3) from the same patient at the beginning (t1, left) and at the end (t3, right) of the study. Quantitative image morphometry was used to assess skeletal muscle index (SMI, upper images) and visceral adipose tissue (VAT, lower images). Over the course of the study, both SMI and VAT visibly decreased in this patient, illustrating progressive loss of muscle mass and visceral fat. Abbreviations: t = time point, ECOG = Eastern Cooperative Oncology Group Performance Status, VAS = Visual Analog Scale, WHO = World Health Organisation.

Adult patients (≥18 years) with a confirmed diagnosis of MM were included if three sequential whole-body CT scans (performed between 2009 and 2024) and complete clinical and laboratory data were available (Fig. 1a). Patients were excluded if they had other malignancies, incomplete CT series (<3 scans), missing clinical or laboratory data, or severe comorbidities unrelated to MM with relevant impact on body composition (e.g., advanced heart failure or neurodegenerative disease).

Of 141 initially identified MM patients with three whole-body CT scans, 86 met all inclusion criteria and were included in the final analysis. The three CT scans were used as reference time points (tCT1–tCT3). The first scan was obtained at initial diagnosis for staging, the second after initiation of systemic therapy during routine disease monitoring, and the third during long-term follow-up according to institutional practice, independent of disease recurrence.

Clinical variables included pain severity assessed by the Visual Analogue Scale (VAS), analgesic use according to the World Health Organization analgesic ladder (WHO I–III), and functional status evaluated using the Eastern Cooperative Oncology Group (ECOG) scale at each time point. Laboratory parameters (serum creatinine, eGFR, and serum albumin) were extracted from electronic medical records at tCT1–tCT3. Baseline 24-h urinary albumin excretion was obtained from electronic records.

Worsening kidney function was defined in analogy to Major Adverse Kidney Event (MAKE) criteria as a ≥25 % decline in eGFR from tCT1 to tCT3, whereas stable kidney function was defined as an eGFR decline <25 % (Maeda et al., 2024; Hofer et al., 2024). Additional stratification was performed according to International Myeloma Working Group (IMWG) criteria for renal impairment (serum creatinine >2 mg/dL or eGFR <40 mL/min) (Dimopoulos et al., 2023).

Albuminuria was categorized according to KDIGO guidelines (A1: <30 mg/24 h; A2: 30–300 mg/24 h; A3: >300 mg/24 h) (Kidney Disease: Improving Global Outcomes (KDIGO) CKD Work Group, 2024). Normoalbuminemia was defined as serum albumin ≥3.5 g/dL and hypoalbuminemia as <3.5 g/dL. A relative serum albumin decline ≥10 % from baseline (tCT1) to follow-up (tCT3) was predefined as clinically relevant, consistent with prior evidence linking such reductions to adverse clinical outcomes (Li et al., 2025).

#### 2.1.1 CT morphometry

CT-morphometric frailty parameters, including skeletal muscle index (SMI), paraspinal muscle area index (PSMI), psoas muscle index (PMI), skeletal muscle density (SMD), and visceral adipose tissue (VAT), were quantified at the L3 vertebral level using automated thresholding with Fiji imaging software (Version 2.3.0/1.53q), as previously described (Malle et al., 2021; Chianca et al., 2022; Yao et al., 2024) (Fig. 1b). Muscle tissue was segmented using standardized attenuation thresholds (−29 to +150 HU), indexed to height (cm^2^/m^2^), and SMD was calculated as mean muscle attenuation (HU).

VAT was segmented using thresholds of −190 to −30 HU, and total adipose tissue area at L3 was quantified. All analyses were performed on native low-dose whole-body CT scans without intravenous contrast. CT morphometric analyses were conducted in a blinded manner by a single experienced physician to ensure analytical consistency.

### 2.2 Statistical analysis

Statistical analyses and graphical visualization were performed using GraphPad Prism (v10.2.2). Differences in CT-morphometric frailty parameters, pain scores, analgesic use, and ECOG status across time points were assessed using Friedman tests with Bonferroni-corrected post hoc comparisons. Data are presented as mean ± standard deviation., and p-values <0.05 were considered statistically significant.

For multivariable analyses, longitudinal changes in body composition were expressed as percentage change in SMI and VAT between baseline and follow-up CT scans. Multivariable linear regression models were used to identify independent predictors of muscle and adipose tissue loss, with percentage change in SMI and VAT as continuous dependent variables. eGFR and albuminuria were included as continuous predictors, while age, sex, body mass index, treatment regimen (IMID therapy), baseline International Staging System (ISS) stage, and cytogenetic risk status (high vs. standard risk) were included as covariates in all models. Regression coefficients (β) with 95 % confidence intervals and two-sided p-values are reported.

## 3 Results

### 3.1 Baseline patient characteristics

The patient demographics and eGFR at baseline are summarized in Table 1, further patient characteristics are summarized in Table 2 and Supplementary Table 1. 86 Patients with diagnosed MM that underwent 3 CT scans with a mean follow-up of 3,2 years were included in this study. The mean age of the study population was 71 ± 11 years. The mean eGFR was 58.2 (14.6) ml/min at baseline. No patient had an eGFR <15 ml/min or required dialysis at baseline. Over the disease course the mean highest albuminuria was 179.1 ± 91.4 mg/24h, with 54 patients (63 %) having an albuminuria of 0–30 mg/24h, 28 patients (33 %) with 30–300 mg/24h, and 4 patients (4 %) with >300 mg/24h. Mean serum albumin was 34.8 ± 6.6 g/l, with 47 % of patients having reduced serum albumin below the local laboratory cutoff (<35 g/l) at baseline. Baseline CT morphometric parameters of sarcopenia and adipose tissue are listed in Table 2. The median ECOG score was 1.0 (range: 0.9) at baseline and was no different between groups (Supplementary Figure 1a). Pain assessment as measured by VAS was 2.0 (range: 6.0), analgesic consumption as measured by WHO scale was 1.0 (range: 7.0) at baseline. Both VAS and WHO did not differ between groups at baseline (Supplementary Fig. 1b–c).

**Table 1:** – Patient demographics and estimated glomerular filtration rate (eGFR) at baseline.

Characteristic	Value
Total Patients (n)	86
Gender	
Female	38
Male	48
Mean Age (years)	
Female	71.4
Male	70.8
BMI (kg/m^2^)	23.1
eGFR (ml/min) distribution (n = male patients)	
≥60	27
45-59	13
30-44	6
15-29	2
<15	0
eGFR (ml/min) distribution (n = female patients)	
≥60	20
45-59	9
30-44	5
15-29	4
<15	0

Table 1 I Patient Demographics and eGFR at Baseline.Table 1 shows the basic patient demographics and eGFR values at baseline.

**Table 2:** – Patient characteristics throughout the study.

Characteristic	tCT1 (Mean/SD)	tCT2 (Mean/SD)	tCT3 (Mean/SD)
eGFR	58.2 (14.6)	51.6 (15.2)	39.1 (16.8)
Male	59.2 (13.7)	54.2 (14.9)	39.0 (16.5)
Female	56.9 (15.8)	48.3 (16.4)	39.3 (17.2)
Serum Albumin (g/l)	34.8 (6.6)	33.5 (4.7)	31.6 (6.3)
Male	34.1 (7.6)	33.2 (5.0)	31.2 (6.8)
Female	35.2 (5.6)	34.1 (4.3)	32.5 (7.2)
Skeletal Muscle Index (SMI) [cm^2^/m^2^]	43.0 (6.9)	36.1 (7.2)	32.7 (6.6)
Male	48.9 (6.8)	41.2 (5.8)	38.9 (8.1)
Female	37.1 (7.3)	30.9 (8.6)	26.5 (4.2)
Paraspinal Muscle Index (PSMI) [cm^2^/m^2^]	16.5 (0.5)	14.6 (0.7)	13.4 (0.8)
Male	16.2 (0.5)	14.4 (0.6)	12.9 (0.8)
Female	16.9 (0.6)	14.9 (0.9)	14.1 (0.8)
Psoas Muscle Index (PMI) [cm^2^/m^2^]	2.6 (0.4)	2.0 (0.3)	1.7 (0.5)
Male	2.8 (0.4)	2.2 (0.3)	1.9 (0.3)
Female	2.4 (0.3)	1.7 (0.3)	1.5 (0.6)
Skeletal Muscle Density (SMD) [HU]	39.5 (8.6)	34.2 (9.1)	30.1 (9.4)
Male	40.2 (8.2)	34.4 (8.9)	31.8 (10.8)
Female	38.9 (9.4)	34.1 (10.5)	28.9 (8.9)
Visceral Adipose Tissue (VAT) [cm^2^]	89.3 (9.2)	76.7 (9.5)	63.9 (8.4)
Male	115.3 (10.7)	100.2 (9.1)	85.3 (9.9)
Female	63.2 (7.1)	53.1 (9.9)	42.6 (7.4)
ECOG	1 (0,9)	1 (4)	2 (3)
VAS	2 (6)	4 (9)	6 (9)
Male	2.2	4.1	6.2
Female	1.8	3.9	5.9
WHO	1 (7)	1 (3)	3 (3)
Male	1.0	1.0	2.8
Female	1.0	1.0	3.1

Table 2 I Patient Characteristics Throughout the Study. Table 2 shows the CT-morphometric parameters as well as clinical patient parameters over the disease course.

### 3.2 Analysis of muscle and adipose tissue changes in relation to kidney function in sequential assessments

**Fig. 2:**
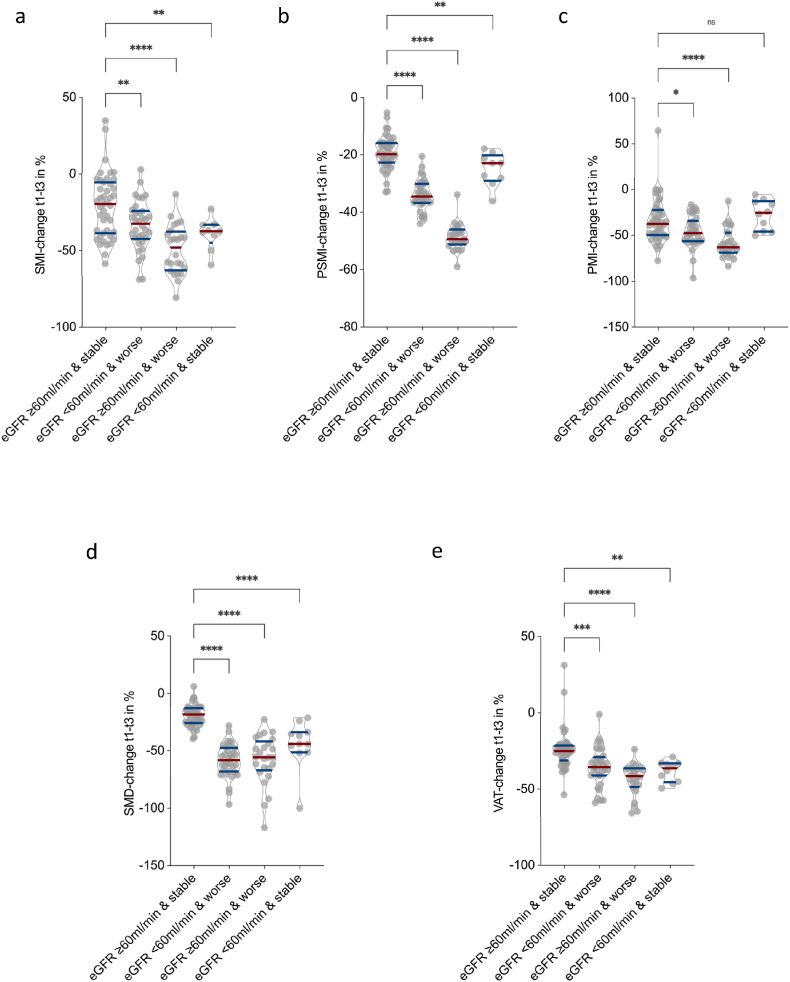
**| Changes in CT-morphometrics between time points 1 and 3 in relation to eGFR throughout the study. a.** Compared to normal and stable kidney function, significant decreases in SMI were observed in patients with decreased eGFR at baseline as well as worsening eGFR irrespective of baseline value. **b.** Compared to normal and stable kidney function, significant decreases in PSMI were observed in all patients with kidney dysfunction. **c.** Compared to normal and stable kidney function, patients with worsening kidney function over the course of disease displayed significant decreases in PMI irrespective of their baseline eGFR. No statistical significance was reached for patients with decreased eGFR at baseline that remained stable over the course of the disease. **d.** Significant decreases in SMD were observed in all patients with kidney dysfunction compared to those with normal and stable kidney function. **e.** All patients with kidney dysfunction exhibited significant decreases in VAT compared to those with normal and stable kidney function. Abbreviations: t = time point, eGFR = estimated glomerular filtration rate, SMI = skeletal muscle index, PSMI = paraspinal muscle index, PMI = psoas muscle index, SMD = skeletal muscle density, VAT = visceral adipose tissue.

Fig. 2 displays the changes between t1 and t3 of each morphometry parameter. Comparisons were performed between patients with an eGFR ≥60 ml/min that remained stable and those with kidney dysfunction.

Patients with an eGFR ≥60 ml/min that remained stable over the course of the study experienced a modest decrease in CT morphometry over time (SMI: −19.3 ± 22.1 %, PSMI: −19.20 ± 5.88 %, PMI: −33.90 ± 24.19 %, SMD: −19.45 ± 9.44 % and VAT: −24.30 ± 13.13 %). Conversely, with kidney dysfunction experienced more accelerated declines in CT morphometry.

Compared to patients with an eGFR ≥60 ml/min that remained stable, those with an eGFR <60 ml/min at baseline that worsened over time experienced a significantly more accelerated decrease in CT morphometry (SMI: −33.4 ± 16.1 % (p < 0.01), PSMI: −33.50 ± 5.81 % (p < 0.0001), PMI: −45.77 ± 16.99 % (p = 0.02), SMD: −58.40 ± 14.64 % (p < 0.0001), VAT: −35.29 ± 12.70 % (p < 0.001)).

Similarly, patients with a baseline eGFR ≥60 ml/min that worsened over the course exhibited more accelerated declines in CT morphometry (SMI: −47.7 ± 17.1 % (p < 0.0001), PSMI: −48.70 ± 4.79 % (p < 0.0001), PMI: −58.09 ± 15.85 % (p < 0.0001), SMD: −58.55 ± 21.32 % (p < 0.0001), VAT: −42.89 ± 10.89 %, (p < 0.0001)).

Ultimately, patients with a baseline eGFR <60 ml/min that remained stable also showed a more pronounced decline in CT morphometry compared to those with normal and stable kidney function (SMI: −36.7 ± 11.2 % (p < 0.01), PSMI: −24.86 ± 5.52 % (p < 0.01), PMI: −27.80 ± 16.47 %, p = 0.44, SMD: −43.47 ± 21.90 % (p < 0.0001), VAT: −38.28 ± 6.89 % (p < 0.01)).

Similar patterns were also observed when stratifying according to IMWG renal impairment criteria. Between tCT1 and tCT3, patients with IMWG-defined renal impairment exhibited more pronounced percentage losses in skeletal muscle quantity and quality as well as adipose tissue, including SMI, PSMI, PMI, SMD, and VAT, compared with patients with preserved renal function (Supplementary Figure 4).

#### 3.2.1 Multivariable analyses of longitudinal body-composition changes

In multivariable linear regression analyses, impaired renal function was independently associated with greater longitudinal loss of skeletal muscle mass and visceral adipose tissue (Supplementary Tables 2 and 3). For SMI, lower eGFR (per 10 ml/min decrease; β −4.8, 95 % CI −6.9 to −2.7; p < 0.001) and higher albuminuria (β −6.1, 95 % CI −9.4 to −2.8; p = 0.01) were both independently associated with greater muscle loss. In addition, high-risk cytogenetics (β −5.5, 95 % CI −9.1 to −2.8; p = 0.02) and ISS stage III (β −6.9, 95 % CI −11.3 to −2.5; p < 0.01) emerged as significant predictors of SMI decline. Age, sex, BMI, and IMID therapy were not independently associated with SMI change.

Similarly, VAT loss was independently associated with reduced eGFR (β −3.7, 95 % CI −5.5 to −1.9; p < 0.01), increased albuminuria (β −5.1, 95 % CI −8.1 to −2.1; p = 0.03), and high-risk cytogenetics (β −6.3, 95 % CI −10.8 to −1.8; p < 0.001). Higher BMI was associated with attenuated VAT loss (β 0.6, 95 % CI 0.2 to 1.0; p = 0.04), whereas ISS stage III did not reach statistical significance in the VAT model.

### 3.3 Analysis of muscle and adipose tissue changes in relation to baseline albuminuria

**Fig. 3:**
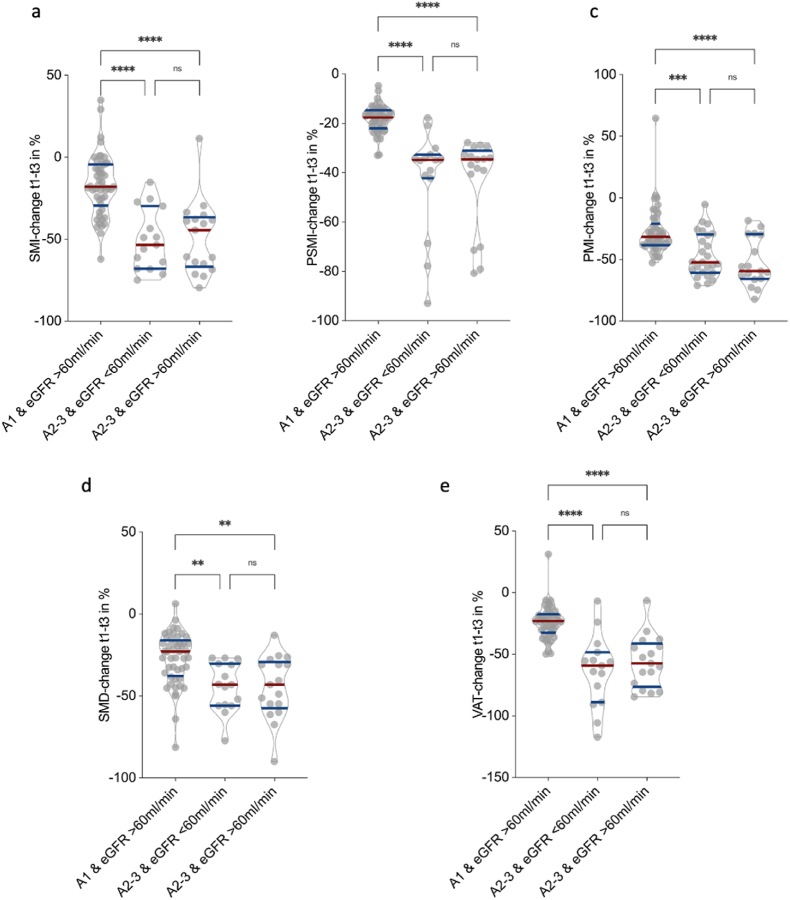
**| Trajectory of CT-morphometrics over the disease course in relation to baseline albuminuria. a.** Patients with an albuminuria ≥30 mg/24h exhibited significantly greater declines in SMI over time compared to those without albuminuria and with normal eGFR. This trend was observed in both patients with eGFR >60 and eGFR <60. No statistically significant differences in SMI dynamics were found between patients with eGFR >60 and < 60 when albuminuria was present (≥30 mg/24h). **b.** Patients with albuminuria ≥30 mg/24h experienced significantly greater reductions in PSMI over time compared to those without albuminuria and with normal eGFR. This pattern was evident in both eGFR >60 and eGFR <60 groups. However, within the albuminuria group (≥30 mg/24h), no significant differences in PSMI decline were observed between patients with eGFR >60 and those with eGFR <60.**c.** Patients with albuminuria ≥30 mg/24h showed a markedly stronger decline in PMI over time compared to individuals without albuminuria and with normal eGFR. This trend was consistent across both eGFR >60 and eGFR <60 subgroups. However, among those with albuminuria (≥30 mg/24h), no significant difference in PMI trajectory was detected between patients with eGFR >60 and eGFR <60. **d.** A significantly more pronounced decrease in SMD over time was observed in patients with albuminuria ≥30 mg/24h compared to those without albuminuria and with normal eGFR. This decline occurred irrespective of whether eGFR was above or below 60. However, within the albuminuria group (≥30 mg/24h), no significant variation in SMD progression was found between patients with eGFR >60 and eGFR <60. **e.** Patients with albuminuria ≥30 mg/24h exhibited a significantly steeper reduction in VAT over time compared to those without albuminuria and with normal eGFR. This effect was seen in both eGFR >60 and eGFR <60 groups. However, among individuals with albuminuria (≥30 mg/24h), VAT decline did not differ significantly between those with eGFR >60 and those with eGFR <60. Abbreviations: t = time point, A = albuminuria grading, eGFR = estimated glomerular filtration rate, SMI = skeletal muscle index, PSMI = paraspinal muscle index, PMI = psoas muscle index, SMD = skeletal muscle density, VAT = visceral adipose tissue.

Fig. 3 illustrates the relationships between baseline albuminuria and CT morphometry over time. The comparative analysis included three patient groups: those with no albuminuria (A1) and an eGFR ≥60 ml/min, those with an albuminuria ≥30 mg/24h (A2-3) and an eGFR <60 ml/min and those with albuminuria ≥30 mg/24h (A2-3) and an eGFR ≥60 ml/min.

Patients with an A1 albuminuria and an eGFR ≥60 experienced a modest decline in CT morphometry (SMI: of −16.11 ± 18.22 %, PSMI: −19.01 ± 5.04 %, PMI: −26.90 ± 18.11 %, SMD: −28.15 ± 16.19 %, VAT: −23.86 ± 13.58 %). In comparison, those with an A2-3 albuminuria and an eGFR <60 ml/min showed a significantly more accelerated decline in CT morphometry (SMI: −52.21 ± 17.66 % (p < 0.0001), PSMI: −44.50 ± 17.15 % (p < 0.0001), PMI: −45.99 ± 17.09 % (p < 0.001), SMD: −44.10 ± 13.91 % (p < 0.01), VAT: −62.09 ± 28.44 % (p < 0.0001)). Similarly, those with an A2-3 albuminuria and an eGFR ≥60 ml/min also displayed a significantly more accelerated loss in CT morphometry compared to patients with A1 albuminuria (SMI: −47.01 ± 21.29 % (p < 0.0001), PSMI: −41.28 ± 21.01 % (p < 0.0001), PMI: −53.44 ± 18.55 % (p < 0.0001), SMD: −44.54 ± 19.27 % (p < 0.01), VAT: −56.06 ± 21.33 % (p < 0.0001)).

#### 3.3.1 Influence of serum albumin at baseline on CT-morphometric assessments

**Fig. 4:**
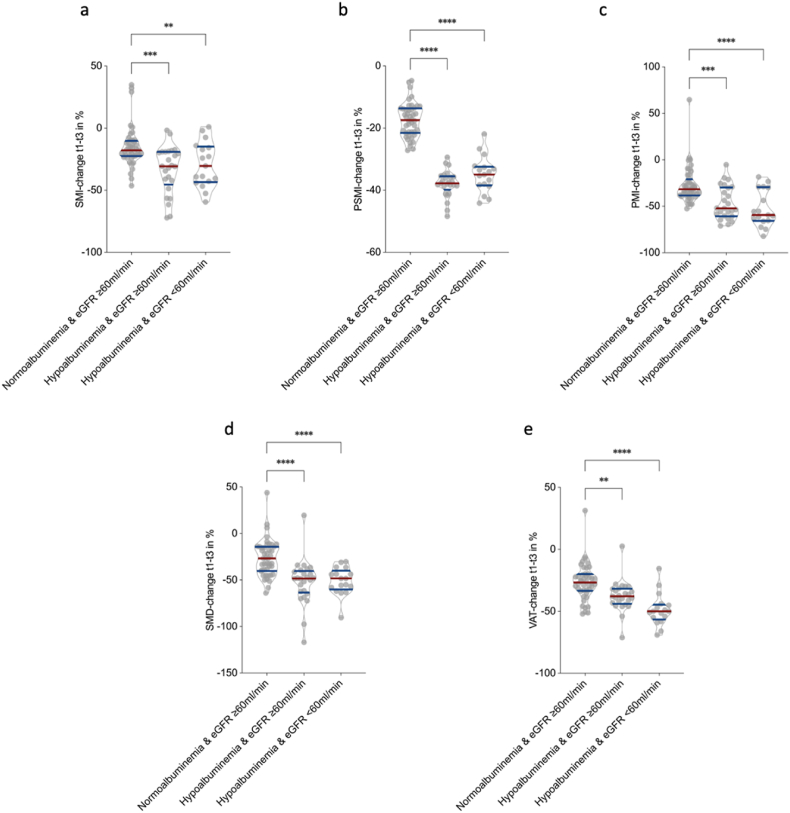
**| Impact of serum albumin levels at baseline on CT-morphometrics throughout the course of the study. a.** Patients with serum albumin <3.5 g/dL showed a significantly greater decrease in SMI over time compared to those with albumin ≥3.5 g/dL. This difference was observed in both eGFR >60 and eGFR <60 groups. **b.** A significantly greater decline in PSMI was observed in patients with serum albumin <3.5 g/dL compared to those with albumin ≥3.5 g/dL. This trend was present in both eGFR >60 and eGFR <60 groups. **c.** Patients with serum albumin <3.5 g/dL experienced a significantly greater reduction in PMI over time compared to those with albumin ≥3.5 g/dL. This pattern was consistent across both eGFR >60 and eGFR <60 groups. **d.** A significantly larger decrease in SMD was observed in patients with serum albumin <3.5 g/dL compared to those with albumin ≥3.5 g/dL. This finding was present in both eGFR >60 and eGFR <60 groups. **e.** Patients with serum albumin <3.5 g/dL showed a significantly greater decline in VAT over time compared to those with albumin ≥3.5 g/dL. This trend was observed in both eGFR >60 and eGFR <60 groups. Abbreviations: t = time point, eGFR = estimated glomerular filtration rate, SMI = skeletal muscle index, PSMI = paraspinal muscle index, PMI = psoas muscle index, SMD = skeletal muscle density, VAT = visceral adipose tissue.

Fig. 4 illustrates the correlations between serum albumin levels at baseline and CT morphometry (SMI, PMI, PSMI, SMD, and VAT) over time.

Patients with a normal serum albumin and an eGFR ≥60 ml/min showed a moderate drop in CT morphometry across all parameters (SMI: −15.09 ± 16.20 %, PSMI: −17.38 ± 5.50 %, PMI: −27.16 ± 19.51 %, SMD: −26.40 ± 19.77 %, VAT: −26.49 ± 14.73 %). In comparison, patients with serum albumin <3.5 g/dL and an eGFR ≥60 ml/min experienced a significantly greater declines across all CT morphometry parameters (SMI: −33.35 ± 19.09 % (p < 0.001), PSMI: −37.91 ± 4.35 % (p < 0.0001), PMI: −46.51 ± 17.97 % (p < 0.001), SMD: −52.05 ± 24.44 %, (p < 0.0001), VAT: −37.76 ± 12.45 % (p < 0.01)). Similarly, patients with serum albumin <3.5 g/dL and an eGFR <60 ml/min also showed significantly accelerated CT morphometry declines (SMI: −29.22 ± 18.11 % (p < 0.01), PSMI: −34.92 ± 5.80 % (p < 0.0001), PMI: −52.37 ± 19.65 % (p < 0.0001), SMD: −50.97 ± 14.92 % (p < 0.0001), VAT: −48.72 ± 12.98 % (p < 0.0001)).

##### 3.3.1.1 Influence of serum albumin dynamics on CT-morphometric assessments

**Fig. 5:**
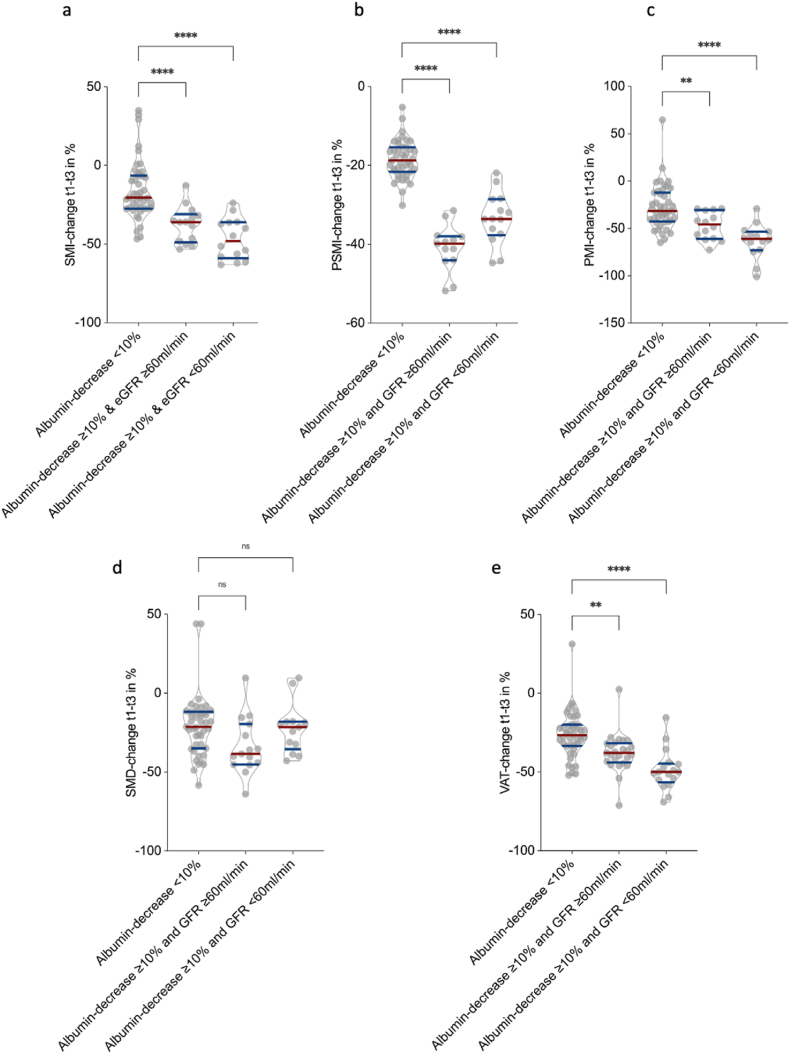
**| Influence of serum albumin trajectory on CT-morphometrics over the course of the study. a.** Patients with an albumin decrease of ≥10 % showed a significantly greater decline in SMI compared to those with an albumin decrease of <10 %, both in the eGFR >60 and eGFR <60 groups. **b.** A significantly greater reduction in PSMI was observed in patients with an albumin decrease of ≥10 % compared to those with an albumin decrease of <10 %, in both eGFR >60 and eGFR <60 groups. **c.** Patients with an albumin decrease of ≥10 % experienced a significantly greater decline in PMI compared to those with an albumin decrease of <10 %, regardless of eGFR status (>60 or <60). **d.** No statistically significant difference in SMD was observed between patients with an albumin decrease of ≥10 % and those with an albumin decrease of <10 %, in either eGFR >60 or eGFR <60 groups. **e.** Between patients with an albumin decrease of ≥10 % and those with an albumin decrease of <10 %, in both eGFR a statistically significant difference in VAT was detected. Abbreviations: t = time point, eGFR = estimated glomerular filtration rate, SMI = skeletal muscle index, PSMI = paraspinal muscle index, PMI = psoas muscle index, SMD = skeletal muscle density, VAT = visceral adipose tissue.

The associations between longitudinal changes in serum albumin and CT morphometrics (SMI, PMI, PSMI, SMD, and VAT) are presented in Fig. 5. Patients were stratified into three groups: those with a stable serum albumin over the course of the study and an eGFR ≥60 ml/min and those with a decrease in serum albumin ≥10 %, further subdivided into an eGFR ≥60 ml/min and an eGFR <60 ml/min.

Patients with a stable serum albumin demonstrated a modest decline in all CT morphometry parameters (SMI: 15.73 ± 18.84 %, PSMI: −18.45 ± 4.83 %, PMI: −27.50 ± 23.48 %, SMD: −20.93 ± 19.62 %, VAT: −26.49 ± 14.73 %). Conversely, those with a decrease in serum albumin and an eGFR ≥60 ml/min as well as an eGFR <60 ml/min displayed accelerated CT morphometry declines across all parameters (SMI: −37.49 % ± 11.57 % and −46.58 ± 13.12 % (both p < 0.0001), PSMI: −40.86 ± 5.89 % and −33.43 ± 6.60 % (both p < 0.0001), PMI: (47.03 ± 14.55 % (p < 0.01) and −63.15 ± 18.34 (p < 0.0001), SMD: −33.67 ± 17.95 % (p = 0.54) and −22.21 ± 16.02 % (p = 0.32), VAT: −37.76 ± 12.45 % (p < 0.01) and −48.72 ± 12.98 % (p < 0.0001).

### 3.4 Influence of kidney dysfunction on clinical function, pain and analgesic consumption in myeloma patients over the disease course

Patients were stratified into four groups according to kidney function: group 1, eGFR ≥60 mL/min and decline <25 %; group 2, eGFR <60 mL/min and decline <25 %; group 3, eGFR ≥60 mL/min and decline ≥25 %; group 4, eGFR <60 mL/min and decline ≥25 %. Changes in ECOG-score, VAS, and WHO analgesic consumption were assessed at t1-3.

**Fig. 6:**
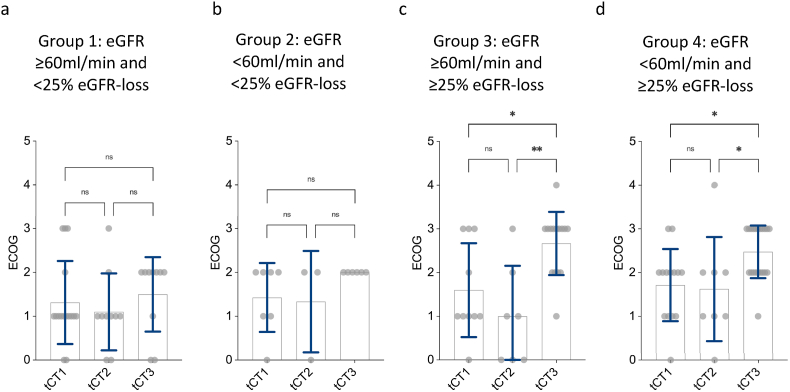
**| Impact of kidney dysfunction on ECOG scores throughout the course of the study. a.** Patients with normal eGFR that remained stable did not experience a change in ECOG score over the course of the study. **b.** Patients with an decreased eGFR that remained stable did not experience a change in ECOG score. **c.** Patients with a normal eGFR at baseline that increased ≥0.3 mg/dl over the course of the study showed a significant increase in ECOG scores between time points 1 and 3 as well as 2 and 3 but not between 1 and 2. **d.** Similarly, patients with a decreased eGFR at baseline decreased for more than 25 % over the course of the study showed significant increases in ECOG scores between time points 1 and 3 as well as 2 and 3 but not between 1 and 2. Abbreviations: t = time point, eGFR = estimated glomerular filtration rate, ECOG = Eastern Cooperative Oncology Group Performance Status.

Patients without eGFR decline (groups 1 and 2) maintained stable ECOG scores across all time points (median ECOG score: 1), indicating preserved functional status (Fig. 6a and b). In contrast, patients with an eGFR decline ≥25 % (groups 3 and 4) demonstrated significant functional deterioration over time. In group 3, the median ECOG score increased from 1 at t1 to 3 at t3 (t1 vs. t3, p < 0.01; t2 vs. t3, p = 0.02) (Fig. 6c). Similarly, in group 4, the median ECOG score increased from 1 at t1 to 3 at t3 (t1 vs. t3, p = 0.01; t2 vs. t3, p = 0.02) (Fig. 6d).

VAS pain scores increased significantly over time in all groups (Supplementary Fig. 2a–d). From t1 to t3, VAS scores increased from 2.0 to 6.0 in group 1 (p < 0.0001), from 1.5 to 6.5 in group 2 (p < 0.01), and from 2.0 to 6.0 in both group 3 and group 4 (p < 0.0001).

Analgesic consumption likewise increased across all groups (Supplementary Fig. 3a–d). Between t1 and t3, WHO scores increased from 1.5 to 3.0 in group 1 (p = 0.02), from 1.4 to 3.0 in group 2 (p < 0.0001), and from 1.1 to 2.5 in both group 3 and group 4 (p < 0.0001).

### 3.5 Exploratory surgical subgroup analysis

Given the clinical relevance of spinal involvement and surgical decision-making in MM patients, an additional exploratory subgroup analysis of surgically treated patients was performed (Supplementary Table 4). Patients were stratified according to the presence of CT-defined sarcopenia at baseline (tCT1). Sarcopenia was defined using commonly applied sex-specific SMI thresholds (<52.4 cm^2^/m^2^ for men and <38.5 cm^2^/m^2^ for women) (Prado et al., 2008).

The aim of this analysis was to evaluate whether baseline sarcopenia was associated with differences in skeletal-related disease burden, neurological deterioration, postoperative clinical status, and longitudinal sagittal alignment changes in surgically treated MM patients. Overall, 34 surgically treated patients were included, of whom 20 patients (58.8 %) met the criteria for sarcopenia at baseline. No significant differences were observed between sarcopenic and non-sarcopenic patients regarding age, sex distribution, BMI, baseline renal function, albuminuria, or serum albumin levels. Similarly, the prevalence of pathological vertebral fractures, multiple vertebral fractures, kyphoplasty rates, and neurological deterioration according to the ASIA classification did not significantly differ between groups. Despite comparable baseline characteristics and spinal disease burden, sarcopenic patients demonstrated significantly poorer clinical outcomes during follow-up. While ECOG performance status at baseline was similar between groups, sarcopenic patients exhibited significantly worse ECOG scores at tCT3 (median ECOG 2.0 vs. 1.0, p = 0.03). Likewise, pain intensity at final follow-up was significantly greater in sarcopenic patients (median VAS 7.0 vs. 5.0, p = 0.02), accompanied by significantly higher analgesic requirements according to the WHO analgesic ladder (median WHO level 3.0 vs. 2.0, p = 0.04). Interestingly, baseline sarcopenia was also associated with more pronounced progressive sagittal deformity over the disease course. While thoracic kyphosis at baseline did not differ significantly between groups, sarcopenic patients demonstrated significantly greater thoracic kyphosis at tCT3 (53.6° vs. 45.1°, p = 0.04) as well as a significantly larger increase in thoracic kyphosis over time (ΔTK +12.9° vs. +5.7°, p = 0.02). In contrast, lumbar lordosis, Dens-S1 distance, and length of hospital stay did not significantly differ between groups.

Overall, these exploratory findings suggest that baseline sarcopenia in surgically treated MM patients may be associated less with the initial extent of spinal disease itself, but rather with progressive functional deterioration, increased pain burden, higher analgesic requirements, and worsening sagittal spinal alignment during the disease course.

## 4 Discussion

Kidney dysfunction is a key complication of MM (Heher et al., 2013; IMWG Recommendations on Managing Multiple). Primarily affecting the elderly, MM patients may frequently inherit multiple co-morbidities, including pre-existing CKD, complicating the course of disease. Frailty is associated with poor outcomes across multiple scenarios, including oncologic patients, (including MM) and CKD (Goede, 2023; Kennard et al., 2023; Falk et al., 2023; Walston et al., 2018) and CT morphometry has recently emerged as valuable and objective assessment tool in this context (Giri et al., 2025).

In this study, we therefore analyzed the associations of kidney dysfunction with CT morphometry as well as functional patient status in MM patients. Our study yielded several key findings:

First, MM patients with impaired eGFR showed accelerated declines in CT morphometry compared to those with normal eGFR. While this decline was most pronounced in patients with a progressive eGFR-loss, those who displayed an eGFR <60 ml/min without further progressive decline also experienced a significantly accelerated loss compared to those with normal and stable kidney function. Secondly, albuminuria was also significantly associated with progressive decline in all CT-morphometrics, highlighting albuminuria, as a potentially useful novel biomarker for future research in MM. Third, kidney function significantly correlated with functional patient outcomes: while patients with progressive kidney dysfunction displayed significant deterioration in ECOG scores over time, those who maintained a stable kidney function showed no functional decline.

Due to its biology, MM directly contributes to kidney damage and sarcopenia. Patients with MM activity often experience pain, significantly limiting physical activity which may accelerate sarcopenia. Furthermore, MM can damage the kidney directly especially in the presence of high disease activity (Palumbo and Anderson, 2011; Solomon et al., 1991; Musolino et al., 2017). Beyond MM-specific mechanisms, the association between renal dysfunction, albuminuria, and sarcopenia has also been demonstrated in several non-oncologic diseases, particularly chronic kidney disease, diabetes mellitus, and cardiovascular disease. Previous studies, including large cohort studies, have shown that impaired renal function and albuminuria are associated with reduced skeletal muscle mass, decreased muscle strength, frailty, and inferior survival outcomes (Wilkinson et al., 2021). Besides specific pathways for inter-organ crosstalk, chronic inflammation, metabolic acidosis, endocrine dysregulation, malnutrition, mitochondrial dysfunction, and physical inactivity have been proposed as central mechanisms linking kidney dysfunction to progressive muscle wasting (Solagna et al., 2021; Zhang et al., 2025).

Importantly, emerging oncologic evidence suggests that this interaction may be particularly relevant in cancer patients exposed to systemic inflammation, catabolic stress, and multimodal therapies. Multiple myeloma itself represents a unique disease model in which renal dysfunction, osteolytic bone destruction, sarcopenia, and systemic frailty closely interact. In a previous longitudinal CT-based study, a progressive vertebral Hounsfield Unit decline in MM patients was associated with renal dysfunction, hypoalbuminemia, increased pain burden, postural decompensation, and reduced survival, suggesting a close interplay between kidney impairment and progressive musculoskeletal deterioration (Kylies et al., 2026). Similarly, in patients undergoing curative-intent surgery for myxofibrosarcoma, acute kidney injury was independently associated with accelerated sarcopenia progression, visceral fat loss, functional decline, increased postoperative morbidity, and impaired overall survival (Kylies et al., 2025). Together, these findings support the hypothesis that renal dysfunction may contribute to progressive musculoskeletal decline across different oncologic diseases and may represent both a marker and potential driver of systemic frailty.

Although novel nephroprotective therapies such as SGLT2 inhibitors have proven beneficial in other kidney disease settings (Zinman et al., 2015; Heerspink et al., 2020; Perkovic et al., 2024; Bakris et al., 2020; Agarwal et al., 2022; Pitt et al., 2021) their applicability in patients with MM remains uncertain, given the distinct pathophysiology of MM-related kidney injury (Heher et al., 2013; Bridoux et al., 2025). While theoretically intriguing, our findings therefore primarily underscore the clinical relevance of kidney dysfunction itself in MM-associated sarcopenia rather than specific therapeutic implications.

From a clinical perspective, our findings may help identify MM patients at increased risk for progressive frailty, functional decline, and treatment intolerance. Importantly, CT morphometry was not only associated with renal dysfunction itself, but also with deterioration in ECOG performance status, suggesting potential relevance for clinically meaningful patient-centered outcomes. In routine practice, MM patients frequently undergo serial CT imaging for disease monitoring, meaning that opportunistic body-composition assessment could theoretically be integrated into standard care without additional imaging burden or radiation exposure. Patients demonstrating progressive eGFR decline, albuminuria, and accelerated CT-morphometric deterioration may represent a particularly vulnerable subgroup requiring intensified supportive care strategies, including nutritional assessment, physiotherapy, closer monitoring of functional decline, optimization of nephroprotective measures, and careful treatment adaptation in frail individuals. Furthermore, progressive CT-morphometric decline may potentially serve as an additional imaging-based biomarker for identifying patients at increased risk of treatment-related complications, prolonged rehabilitation, or reduced tolerance to intensive systemic therapy. While prospective validation is required before implementation into routine clinical decision-making, our findings support the concept that combining renal parameters with longitudinal CT morphometry may improve biologically informed frailty assessment in MM patients beyond conventional clinical scores alone.

Importantly, our exploratory subgroup analysis of surgically treated MM patients further supports the clinical relevance of CT-defined sarcopenia in spinal MM disease. While baseline sarcopenia was not associated with greater fracture burden, kyphoplasty rates, or neurological deterioration, sarcopenic patients demonstrated significantly worse functional outcomes during follow-up, including poorer ECOG performance status, increased pain intensity, higher analgesic requirements, and more pronounced thoracic kyphosis progression. These findings suggest that CT-defined sarcopenia may reflect reduced biomechanical and functional reserve rather than the initial extent of spinal disease itself. Consequently, opportunistic CT-based body composition assessment may help identify surgically treated MM patients at increased risk for progressive functional decline and postural decompensation during the disease course. However, these exploratory findings require validation in larger prospective cohorts.

Our study has several strengths and limitations. To the best of our knowledge, no previous study has analyzed the impact of kidney dysfunction on CT morphometry in MM patients. Importantly, only limited evidence exists regarding the stratification of MM patients based on progressive versus stable kidney dysfunction, even though CKD may be a prevalent yet underrepresented comorbidity in MM. Furthermore, the role of albuminuria has not been systematically explored in MM, despite its broad use in nephrology-focused care. The longitudinal study design allows for an insightful determination of the trajectory of muscle and fat loss in these patients, providing valuable insights into the progression of sarcopenia in MM patients.

Despite its strength, several limitations must be considered. Our inclusion criterion requiring three sequential whole-body CT scans, potentially enriching the cohort for patients who survived long enough to undergo repeated imaging may predispose for selection bias and the modest sample size and single-center design may limit the generalizability.

Another limitation relates to temporal heterogeneity, as the study period spans multiple therapeutic eras in MM management. Earlier regimens differ from contemporary therapies and may exert distinct metabolic effects. Although most patients in our cohort received IMID-based regimens and treatment regimen was included in the multivariable analyses, residual confounding cannot be fully excluded.

Additionally, the retrospective design limits the ability to establish causality. Although MRI may provide superior soft-tissue characterization and more detailed assessment of intramuscular composition, CT-based morphometry offers substantial practical advantages in MM patients, as whole-body CT imaging is routinely performed during disease staging and follow-up, enabling opportunistic longitudinal body-composition assessment without additional imaging burden. While we showed associations between kidney dysfunction and CT-morphometrics and functional status, the causality remains unclear. Particularly, the question remains whether their association is related to progressive MM, directly affecting muscle and adipose tissue via systemic inflammation and pro-inflammatory cytokines like IL-6 and TNF-α (Akhmetzyanova et al., 2021), as well as kidney function via MM-induced kidney damage (Solomon et al., 1991), or if the impaired kidney function itself negatively impacts body composition. Interestingly, besides patients with progressive decline in kidney function those with impaired kidney function at baseline that remained stable also showed significantly accelerated progression in CT-morphometrics. In the light of previous studies highlighting CKD as a molecular driver of sarcopenia (Puelles and Huber, 2022; Solagna et al., 2021) a direct impact of kidney dysfunction on sarcopenia in MM patients, at least partially, appears possible. However, further prospective and molecular studies are needed.

Another interesting observation is the association between albuminuria and progressive CT-morphometrics. While albuminuria is a known risk factor for cardiovascular events (Gerstein et al., 2001), a frailty in the elderly (Chang et al., 2016), and oncologic patients (Luo et al., 2023), its implications in MM are poorly understood. Our study here may serve as a pilot, encouraging further research.

Despite these limitations, our study provides valuable insights into the relationship between kidney function, muscle and adipose tissue loss in MM patients.

In conclusion, this study links kidney dysfunction, including albuminuria, to accelerated sarcopenia in patients with MM. However, given the retrospective design and associated limitations, these findings require confirmation in larger and ideally prospective studies. In the future, combining renal parameters, including albuminuria, with imaging-derived body composition assessment could help improve risk stratification and supportive care planning in patients with MM.

## Human ethics approval declaration and consent to participate declarations

This retrospective observational study was approved by the Ethics Committee of the Hamburg Medical Association (ethics ID: 2025-300576-WF) and conducted in accordance with the principles of the Declaration of Helsinki. In view of the fact that the patient data that are the subject of the study can no longer be attributed to a human being, the study does not constitute a "research project involving human beings" as defined in Section 9 (2) of the Hamburg Chamber Act for the Medical Professions and also does not fall within the scope of the research projects requiring consultation pursuant to Section 15 (1) of the Professional Code of Conduct for Hamburg Physicians. Therefore, the requirement for informed consent was waived.

## Author contributions

Kylies J, Kylies D designed and conceptualized the research study. Kylies J and Kylies D extracted the clinical data. Kylies J performed the statistical analysis. Kylies D wrote the manuscript. Kylies J and Kylies D generated the figures and tables. Wenzel UO, Huber TB, Weisel K, Viezens L and Leonhardt L-G provided critical review and content-related feedback. Leonhardt L-G oversaw the study as a senior scientist.

## Data access statement

The data underlying this manuscript will be shared upon reasonable request.

## Funding

DK received funding from the UKE Foundation.

## Conflict of interest

None.
