# Supplementary material for: Multiple myeloma with spinal involvement: Renal dysfunction and albuminuria are associated with progressive sarcopenia in a longitudinal CT morphometric study
Source: Brain Spine. 2026 Jun 30;6:106153. doi: 10.1016/j.bas.2026.106153 (PMC13355679; doi:10.1016/j.bas.2026.106153)
Supplement: Multimedia component 1 [file mmc1.docx]

**Supplementary Table 1 - Myeloma-related clinical characteristics of the patient population**

| **Characteristic** | **Total (n=86)** | **IMWG criteria: renal impairment (n=15)** | **IMWG criteria:  no renal impairment (n=71)** |
| --- | --- | --- | --- |
| ISS |  |  |  |
| I | 23 (27%) | 6 (40%) | 17 (24%) |
| II | 38 (44%) | 4 (27%) | 34 (48%) |
| III | 25 (29%) | 5 (33%) | 20 (28%) |
| IgG Type | 46 (53%) | 9 (60%) | 37 (52%) |
| Cytogenetic risk profile |  |  |  |
| low | 42 (49%) | 5 (33%) | 37 (52%) |
| high | 38 (44%) | 6 (40%) | 32 (45%) |
| unknown | 6 (7%) | 4 (27%) | 2 (3%) |
| Steroids received (e.g. dexamethasone) |  |  |  |
| yes | 86 (100%) | 15 (100%) | 71 (100%) |
| no | 0 (0%) | 0 (0%) | 0 (0%) |
| Immunmodulatory drugs |  |  |  |
| Lenalidomide | 61 (71%) | 12 (80%) | 49 (69%) |
| Thalidomide | 15 (17%) | 2 (13%) | 13 (18%) |
| Other | 5 (6%) | 1 (7%) | 4 (6%) |
| None | 5 (6%) | 0 (0%) | 5 (7%) |
| Autologous stem cell transplantation received | 49 (57%) | 12 (80%) | 37 (52%) |

**Supplementary Table 2 – Multivariable linear regression for percentage change in SMI**

| **Variable** | **β 95% Confidence Interval** | **p-value** |
| --- | --- | --- |
| eGFR (per 10ml/min | -4.8 (-6.9 to -2.7) | <0.001 |
| Baseline albuminuria | -6.1 (-9.4 to -2.8) | 0.01 |
| High cytogenetic risk | -5.5 (-9.1 to -2.8) | 0.02 |
| ISS stage III | -6.9 (-11.3 to -2.5) | <0.01 |
| Age (per year) | -0.3 (-0.6 to 0.2) | 0.44 |
| Male sex | 2.4 (-3.2 to 8.0) | 0.51 |
| BMI (per kg/m^2^) | 0.4 (-0.1 to 0.9) | 0.10 |
| IMID therapy | 1.9 (-4.2 to 8.0) | 0.54 |

**Supplementary Table 3 – Multivariable linear regression for percentage change in VAT**

| **Variable** | **β 95% Confidence Interval** | **p-value** |
| --- | --- | --- |
| eGFR (per 10ml/min | -3.7 (-5.5 to -1.9) | <0.01 |
| Baseline albuminuria | -5.1 (-8.1 to -2.1) | 0.03 |
| High cytogenetic risk | -6.3 (-10.8 to -1.8) | <0.001 |
| ISS stage III | -3.3 (1.9 to -5.4) | 0.11 |
| Age (per year) | -1.1 (-1.2 to 0.4) | 0.45 |
| Male sex | 1.6 (-3.1 to 6.3) | 0.50 |
| BMI (per kg/m^2^) | 0.6 (0.2 to 1.0) | 0.04 |
| IMID therapy | 2.5 (-3.5 to 8.5) | 0.41 |

**Supplementary Table 4 - Exploratory surgical subgroup analysis**

| **Parameter** | **Non-sarcopenic at tCT1** | **Sarcopenic at tCT1** | **p-value** |
| --- | --- | --- | --- |
| Patients, n | 14 | 20 | — |
| Age, years | 69.8 ± 9.4 | 72.6 ± 10.8 | 0.31 |
| Female sex, n (%) | 5 (36%) | 9 (45%) | 0.59 |
| BMI, kg/m² | 24.1 ± 3.2 | 22.4 ± 3.8 | 0.12 |
| Baseline eGFR, mL/min | 59.8 ± 13.9 | 55.4 ± 15.8 | 0.36 |
| Baseline albuminuria >30 mg/24 h, n (%) | 5 (36%) | 9 (45%) | 0.58 |
| Baseline serum albumin, g/L | 35.6 ± 5.8 | 33.7 ± 6.4 | 0.28 |
| Pathological vertebral fractures, n (%) | 12 (86%) | 18 (90%) | 0.71 |
| Multiple fractures ≥4, n (%) | 2 (14%) | 4 (20%) | 0.66 |
| Kyphoplasty, n (%) | 8 (57%) | 12 (60%) | 0.87 |
| Instrumented stabilization, n (%) | 14 (100%) | 20 (100%) | — |
| ASIA deterioration, n (%) | 1 (7%) | 2 (10%) | 0.76 |
| ECOG at tCT1 | 1.0 (0–2) | 1.0 (0–2) | 0.84 |
| ***ECOG at tCT3** | 1.0 (0–2) | 2.0 (1–4) | **0.03** |
| VAS at tCT1 | 2.0 (0–5) | 2.5 (0–6) | 0.62 |
| ***VAS at tCT3** | 5.0 (2–8) | 7.0 (4–9) | **0.02** |
| WHO analgesic score at tCT1 | 1.0 (0–2) | 1.0 (0–2) | 0.77 |
| ***WHO analgesic score at tCT3** | 2.0 (1–3) | 3.0 (2–3) | **0.04** |
| Thoracic kyphosis tCT1, ° | 39.4 ± 12.8 | 40.7 ± 13.5 | 0.73 |
| ***Thoracic kyphosis tCT3, °** | 45.1 ± 14.2 | 53.6 ± 15.8 | **0.04** |
| ***ΔThoracic kyphosis tCT1–tCT3, °** | +5.7 ± 7.9 | +12.9 ± 9.4 | **0.02** |
| Lumbar lordosis tCT3, ° | 49.8 ± 11.7 | 47.3 ± 13.2 | 0.49 |
| Dens-S1 distance tCT3, cm | 49.7 ± 3.1 | 48.6 ± 3.8 | 0.34 |
| Length of hospital stay, days | 18.4 ± 7.9 | 22.6 ± 10.3 | 0.15 |

a

b

c


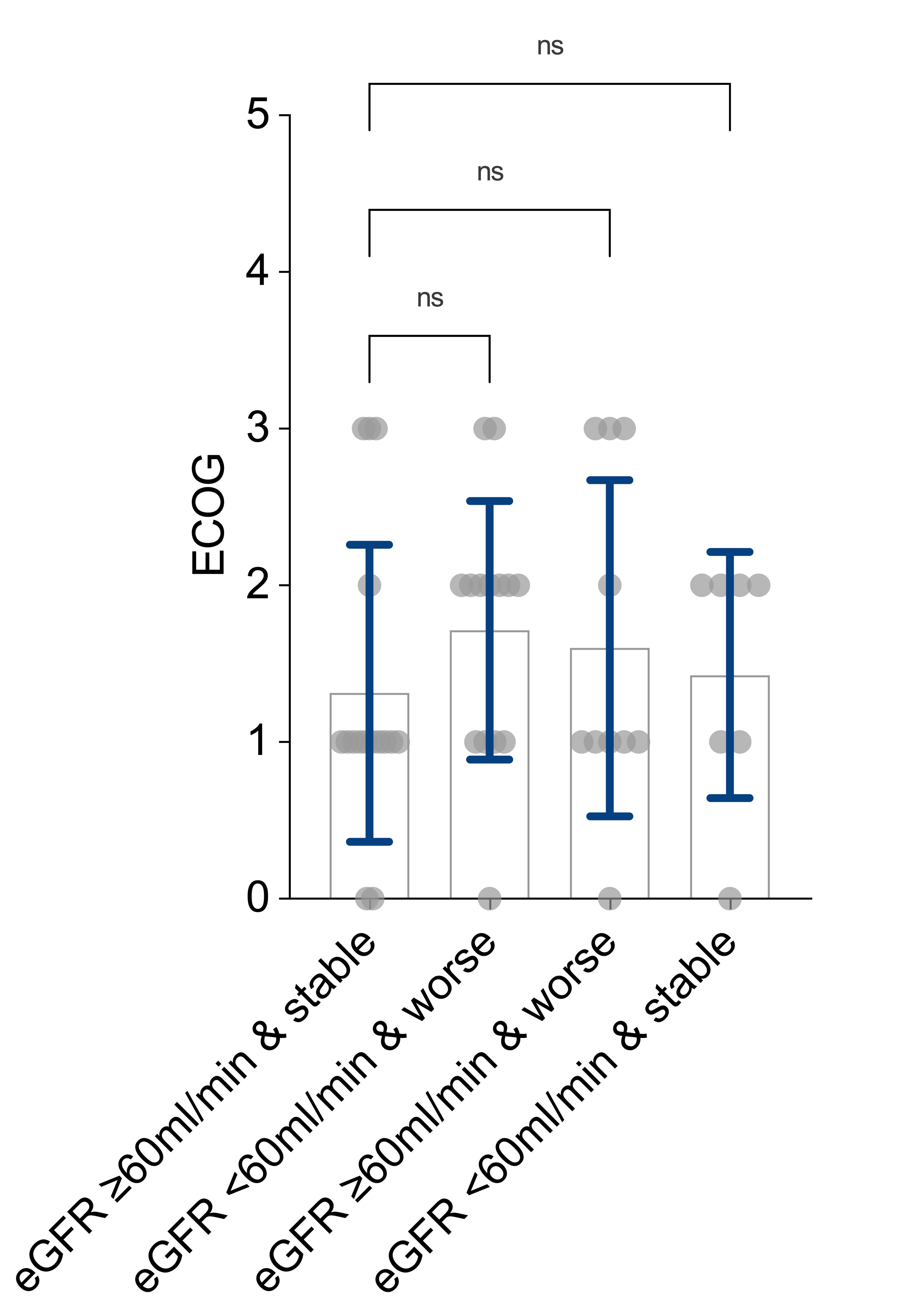

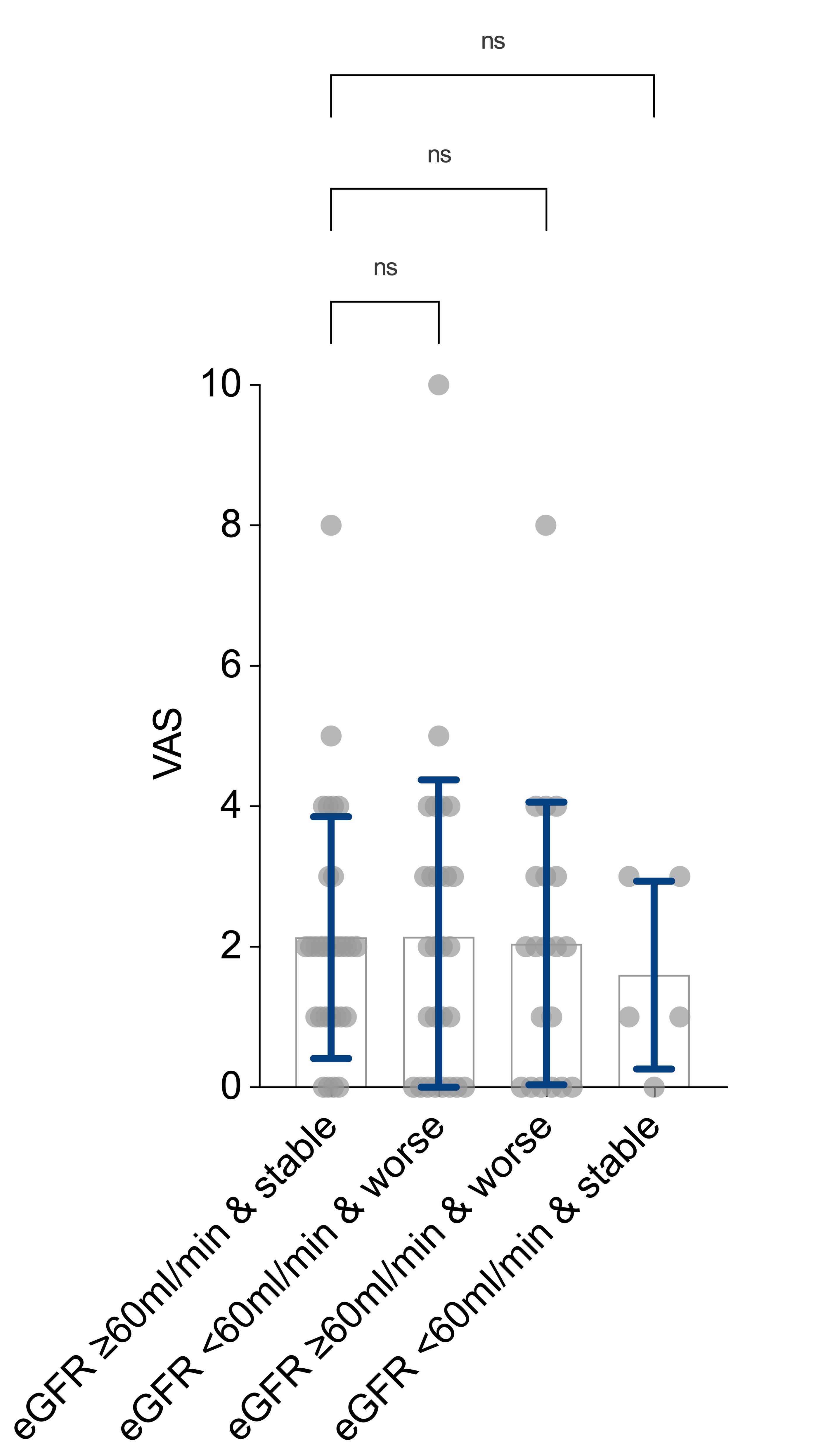

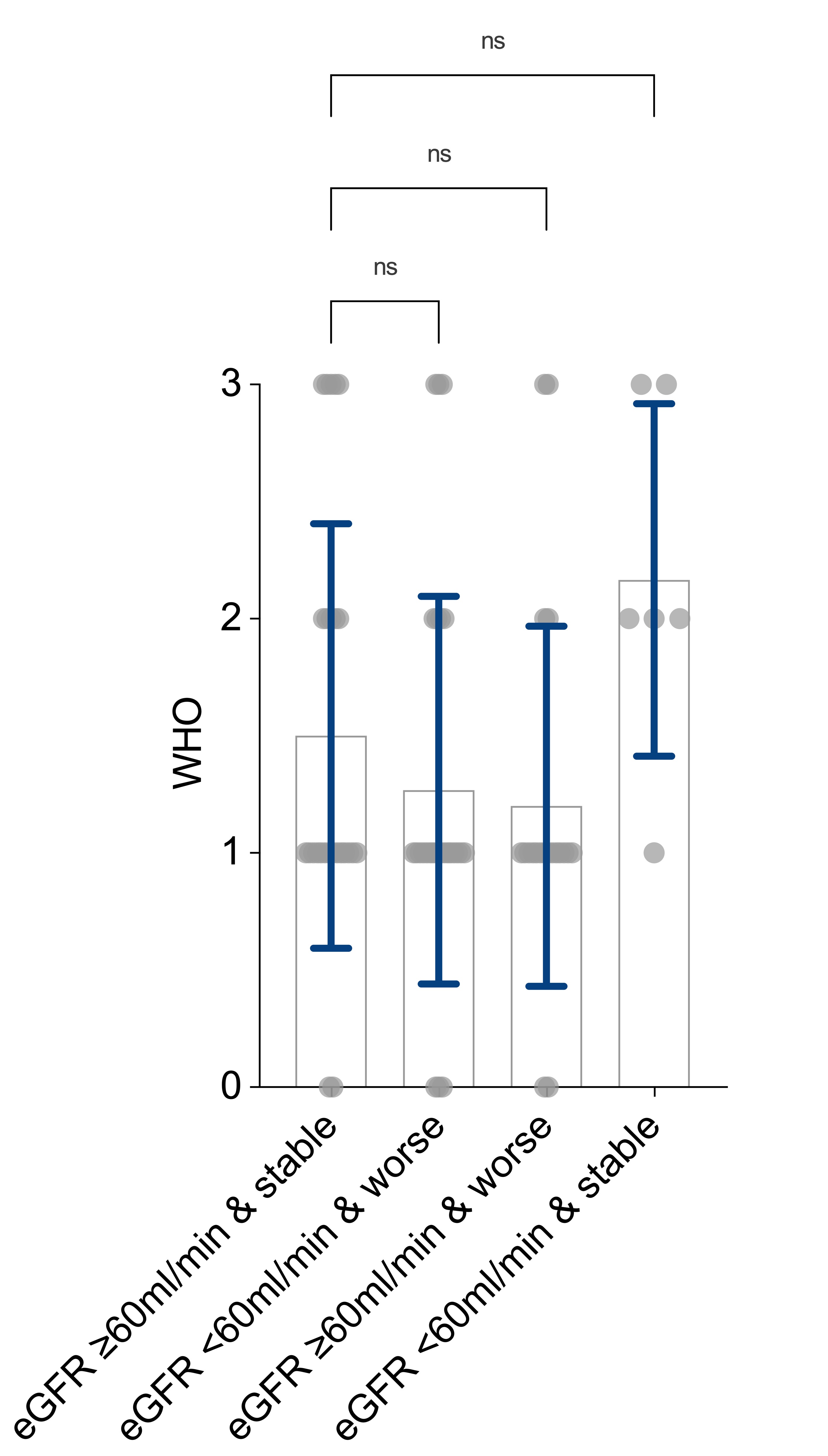


**Supplementary Figure 1 | ECOG, WHO and VAS at baseline. a.** No significant difference in ECOG score was present between patients with a eGFR ≥60ml/min that remained stable over the course of the study compared to other groups with kidney dysfunction. **b.** No significant difference analgesic consumption measured by WHO score was present between patients with normal and stable kidney function compared to patients with kidney dysfunction. **c.** Similarly, no significant differences in pain symptom burden measured by VAS score were present between patients with normal and stable kidney function and to patients with kidney dysfunction. Abbreviations: eGFR = estimated glomerular filtration rate, ECOG = Eastern Cooperative Oncology Group Performance Status, WHO = World Health Organisation, VAS = Visual Analog Scale.


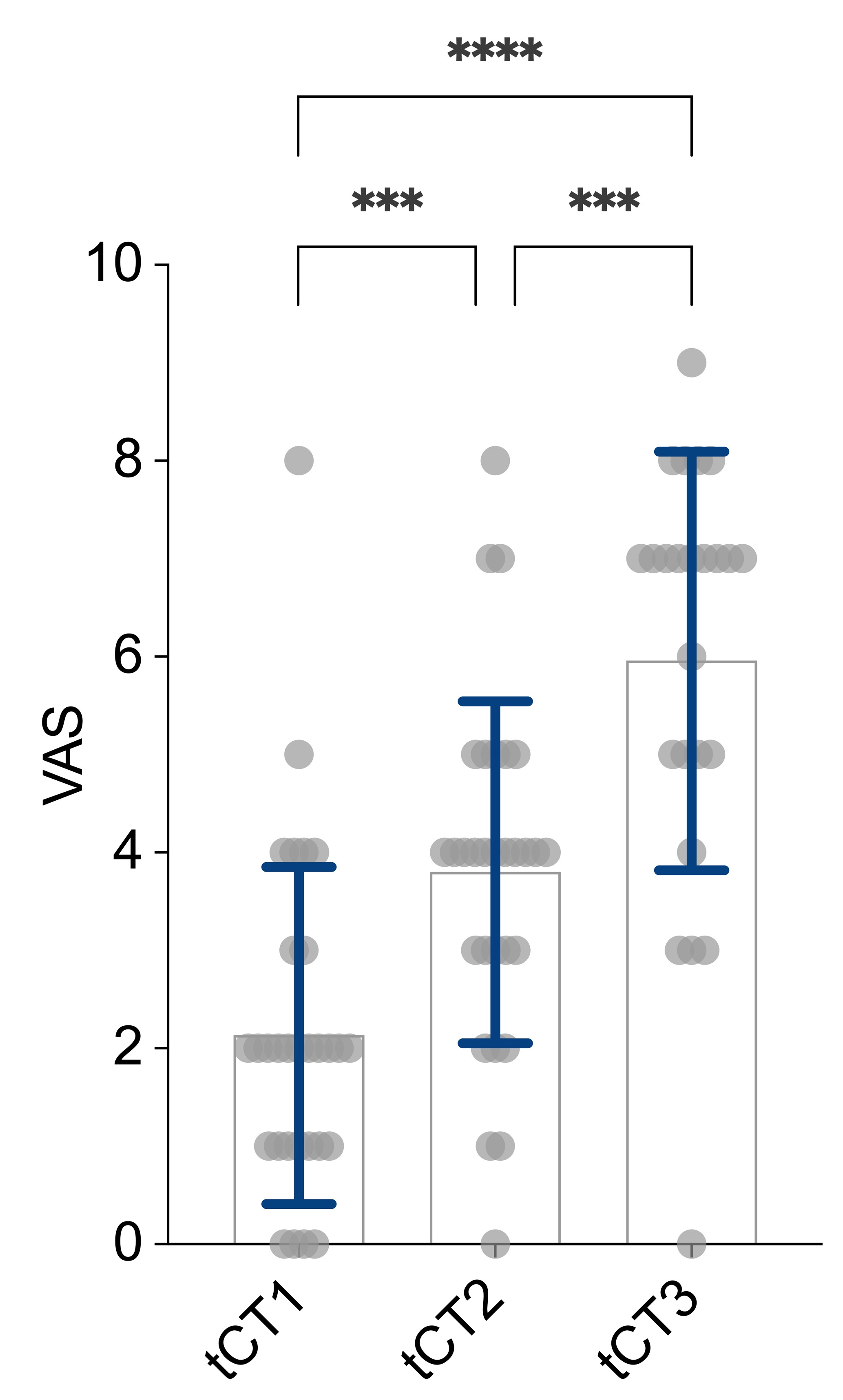

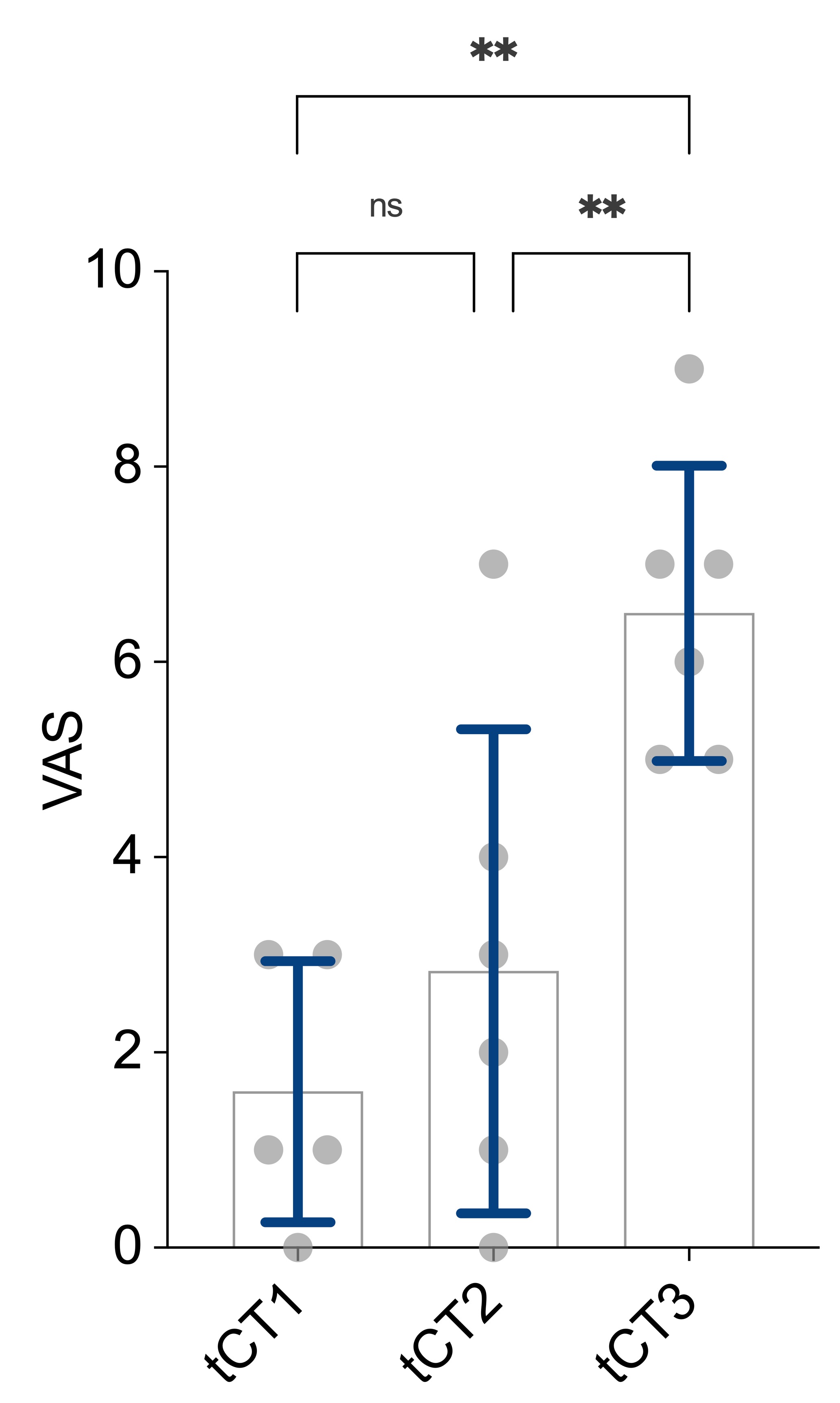

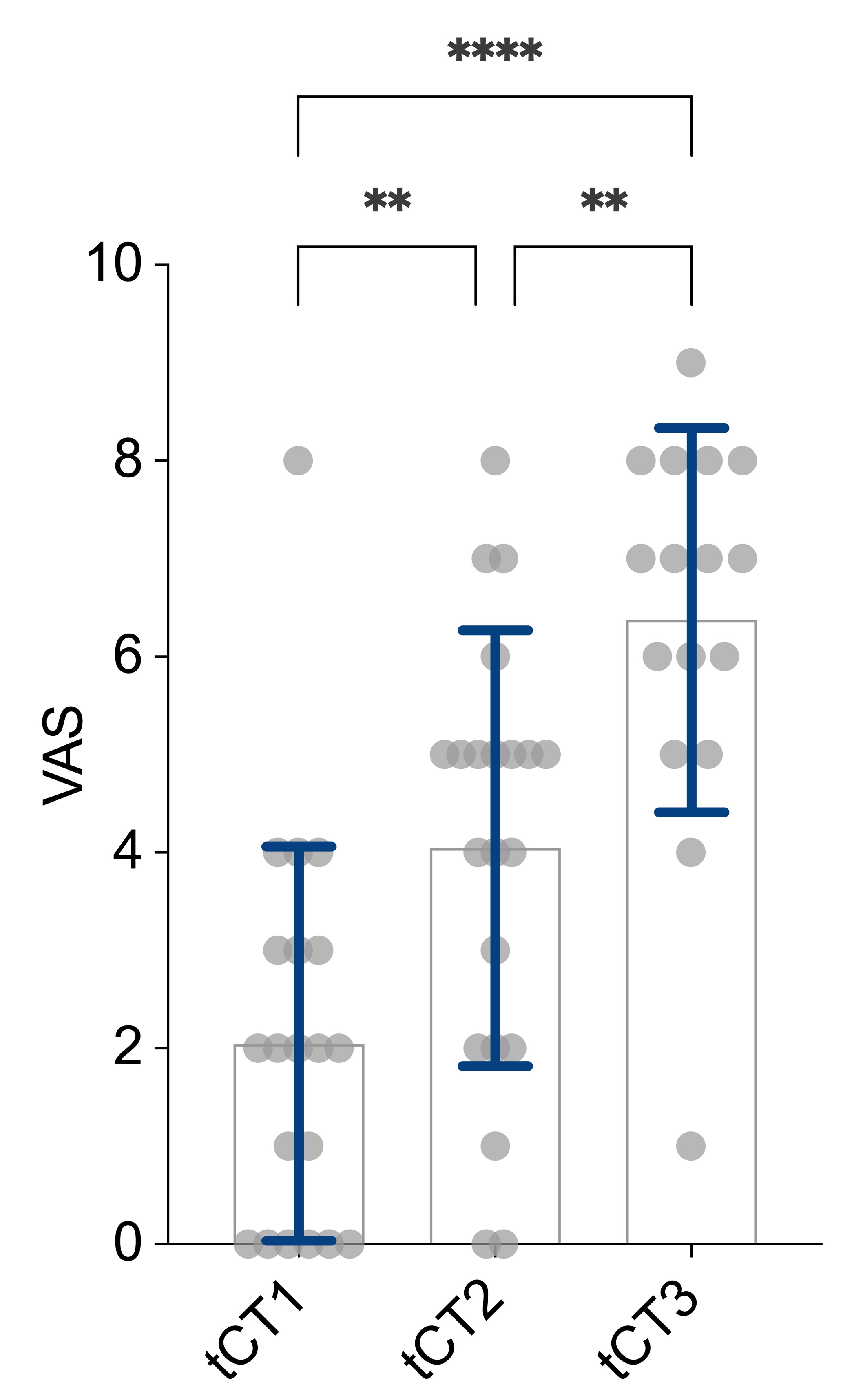

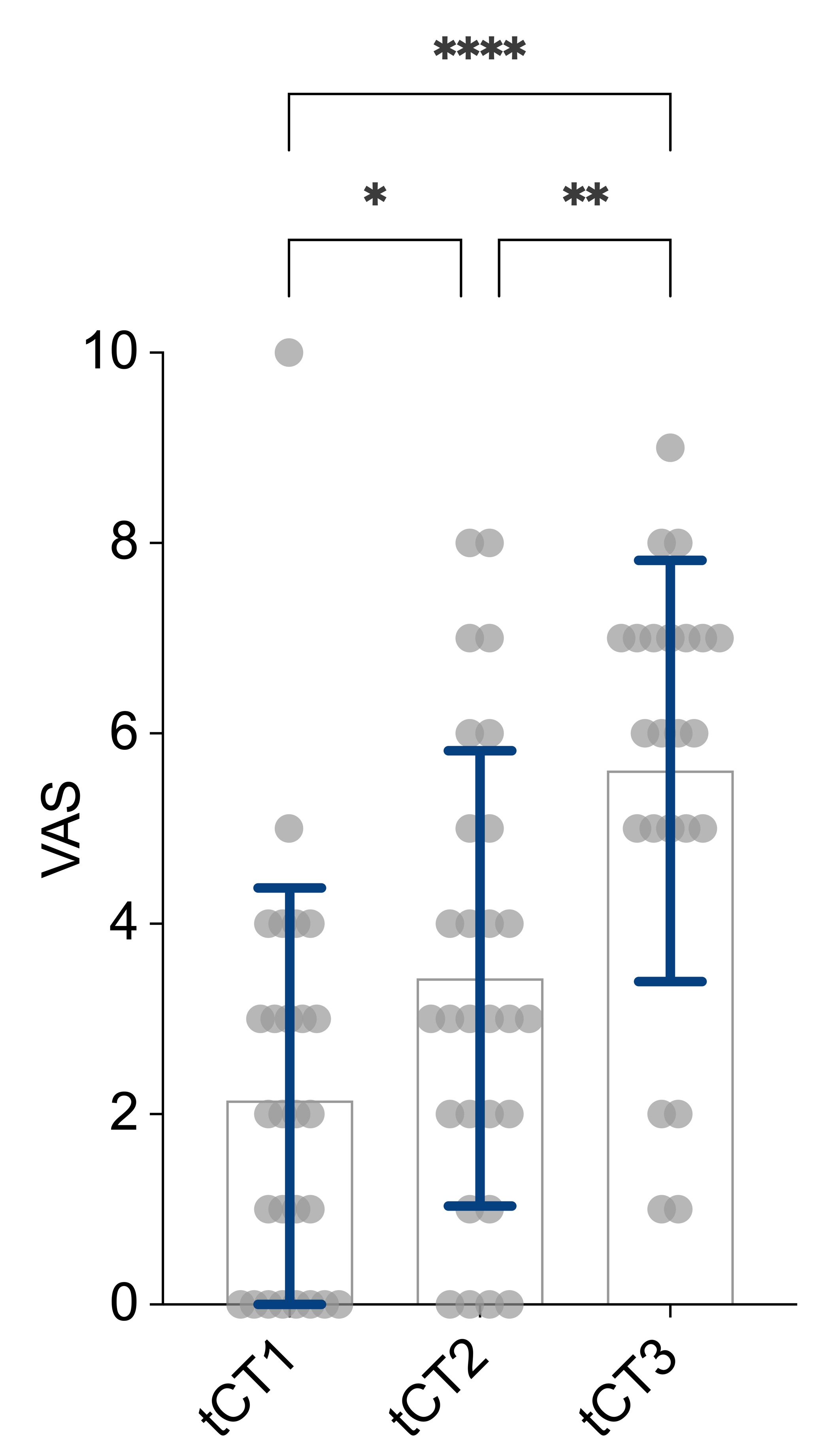


Group 1: eGFR ≥60ml/min and <25% eGFR-loss

Group 3: eGFR ≥60ml/min and

≥25% eGFR-loss

Group 2: eGFR <60ml/min and <25% eGFR-loss

Group 4: eGFR <60ml/min and

≥25% eGFR-loss

**Supplementary Figure 2 | Changes in pain symptom burden measured by VAS over the course of the study. a.** Patients with normal and stable kidney function showed significant progression in pain scale measured by VAS between time points 1 and 2, 1 and 3 as well as 2 and 3. **b.** Patients with decreases eGFR that remained stable over the course of the study showed significant progression in VAS between time points 1 and 3 and 2 and 3 but not between 1 and 2. **c.** Patients with normal eGFR at baseline that worsened over the course of the study displayed significant progression in pain symptom burden as measured by VAS between time points 1 and 2, 1 and 3, and 2 and 3. **d.** Patients with decreased eGFR at baseline that worsened over the course of the study also displayed significant progression in VAS between time points 1 and 2, 1 and 3, and 2 and 3. Abbreviations: t = time point, eGFR = estimated glomerular filtration rate, VAS = Visual Analog Scale.

a

c

b

d


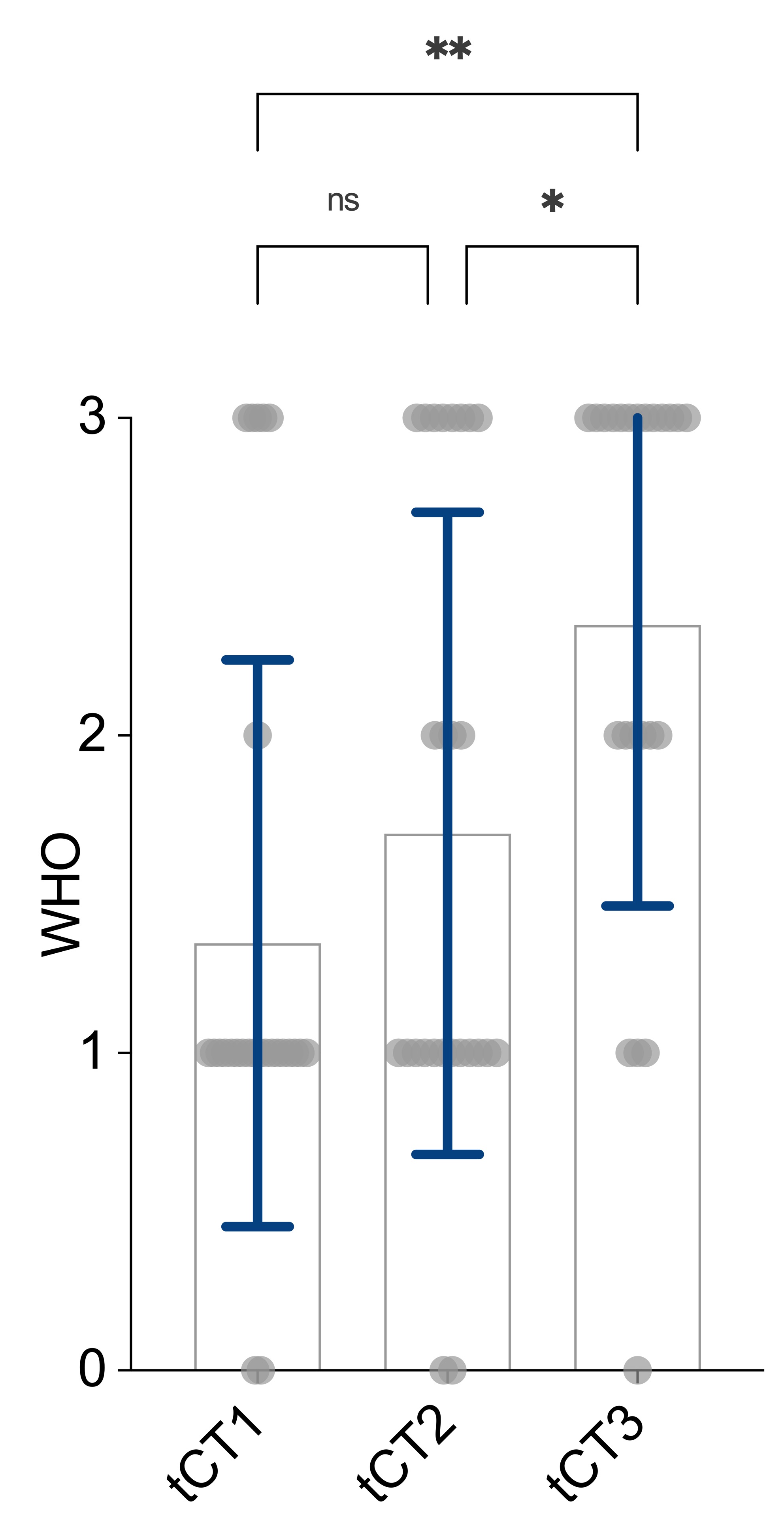

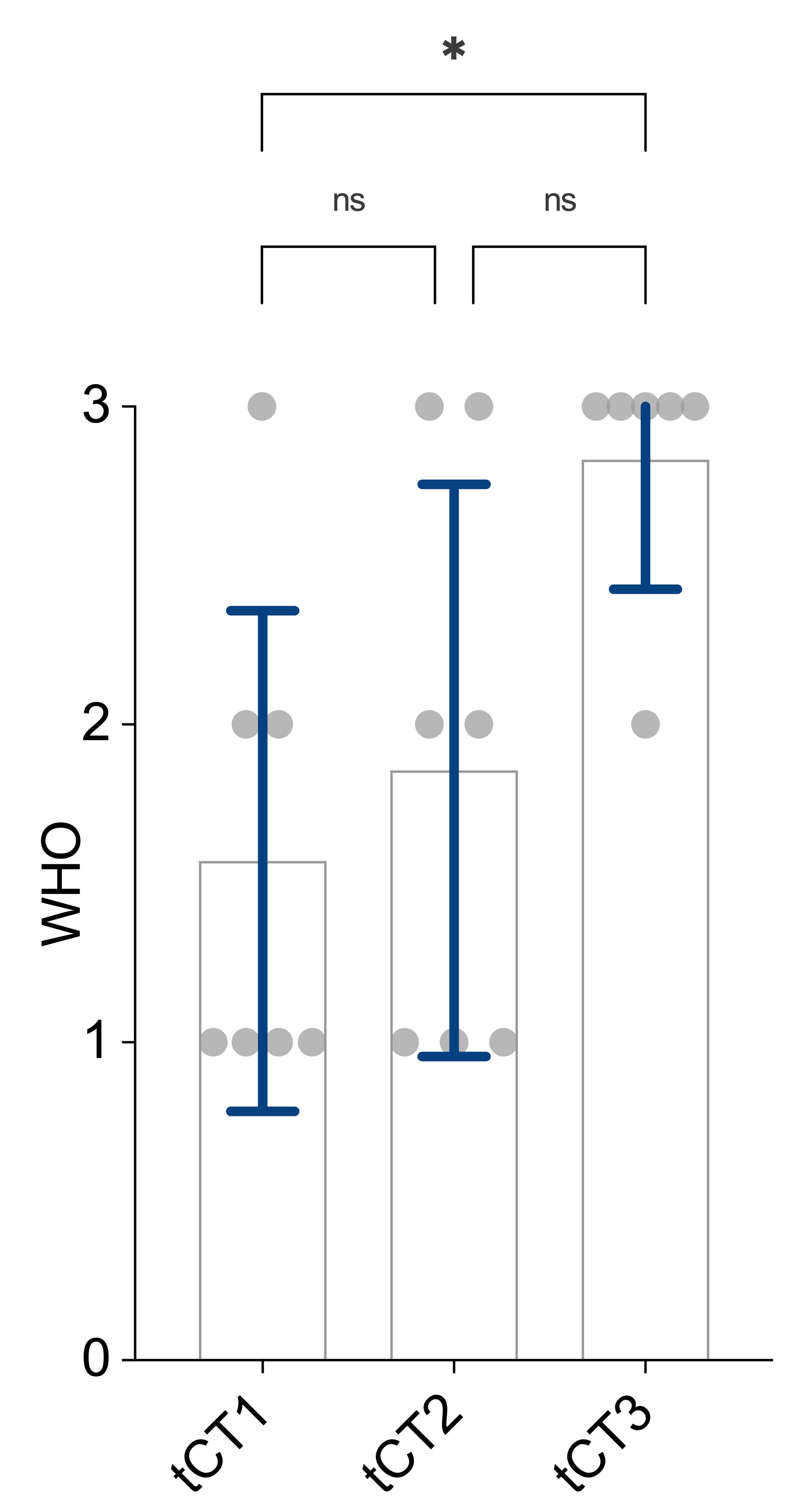

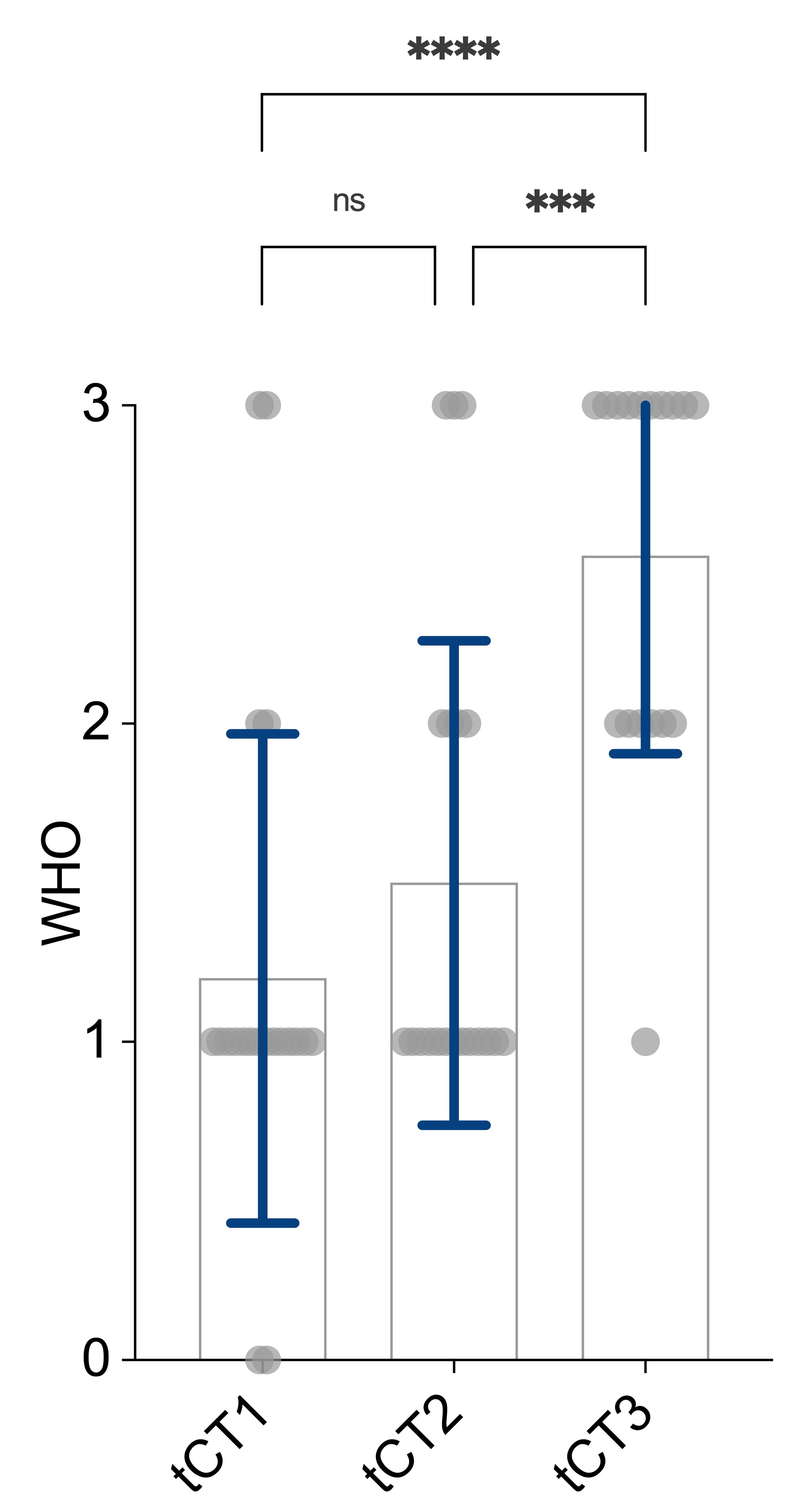

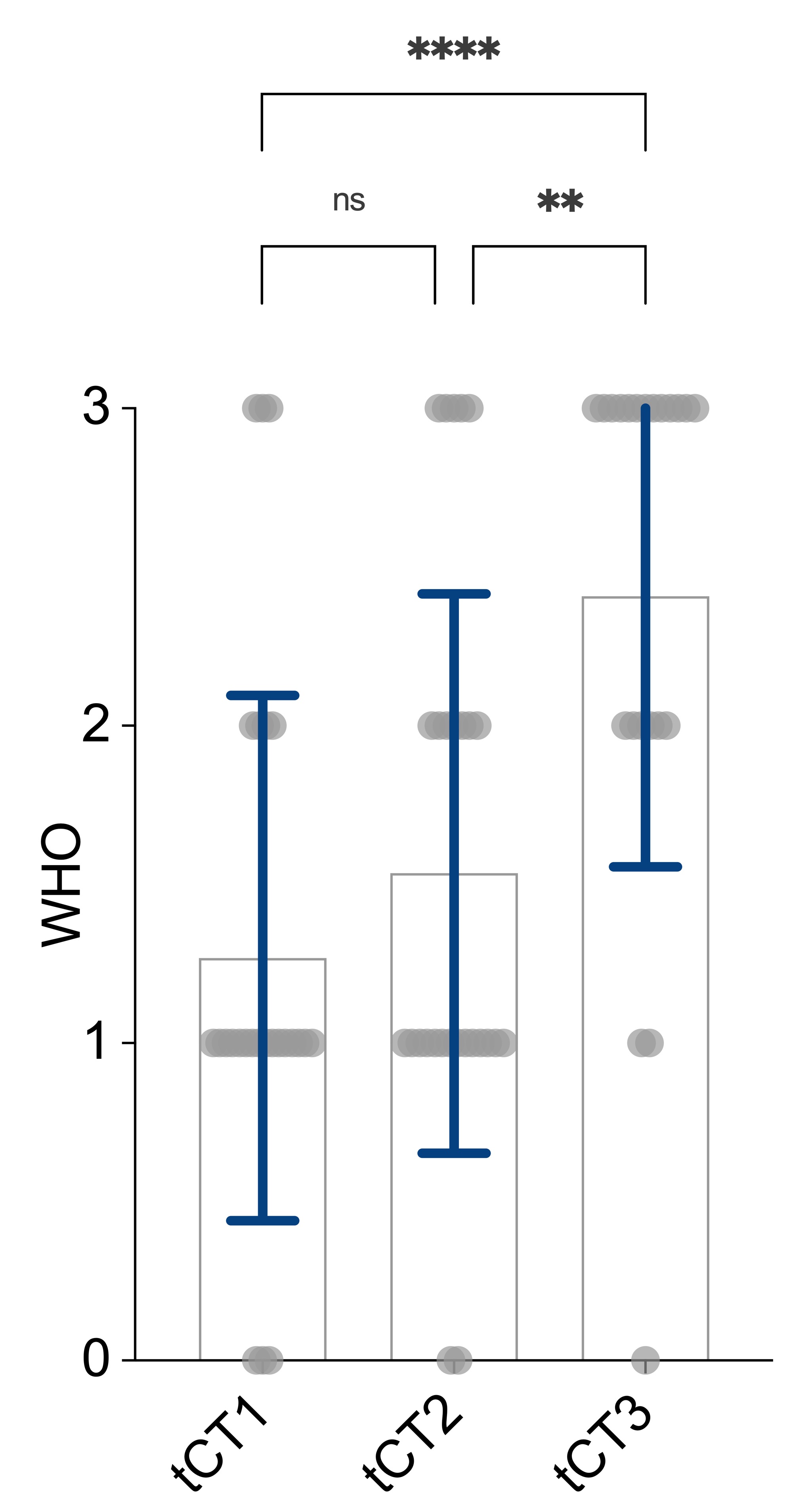


Group 1: eGFR ≥60ml/min and <25% eGFR-loss

Group 3: eGFR ≥60ml/min and

≥25% eGFR-loss

Group 2: eGFR <60ml/min and <25% eGFR-loss

Group 4: eGFR <60ml/min and

≥25% eGFR-loss

**Supplementary Figure 3 | Changes in analgesic consumption measured by WHO-score over the course of the study. a.** Patients with normal and stable kidney function showed significant progression in pain scale measured by WHO-score between time points 1 and 3 as well as 2 and 3. **b.** Patients with decreased eGFR that remained stable showed significant progression in WHO-score between time points 1 and 3 but not between 1 and 2 or 2 and 3. **c.** Patients with normal eGFR at baseline that worsened over the course of the study displayed significant progression in WHO-score between time points 1 and 3 as well as 2 and 3 but not between 1 and 2. **d.** Patients with decreased eGFR at baseline that worsened over the course of the study showed a significant progression in WHO-score between time points 1 and 3 as well as 2 and 3 but not between 1 and 2.

Abbreviations: t = time point, eGFR = estimated glomerular filtration rate, WHO = World Health Organisation, VAS = Visual Analog Scale.

a

e

d

b

c


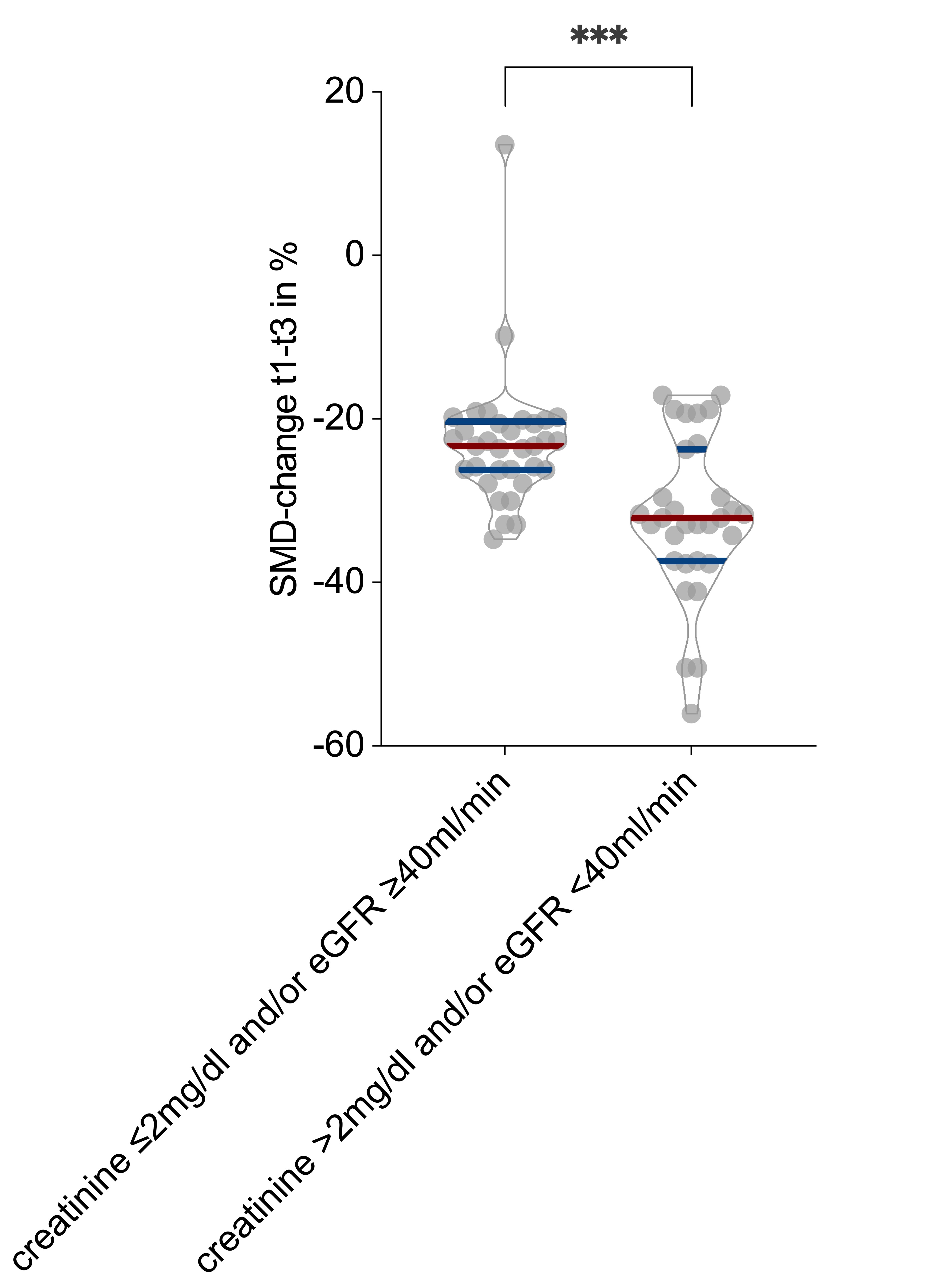

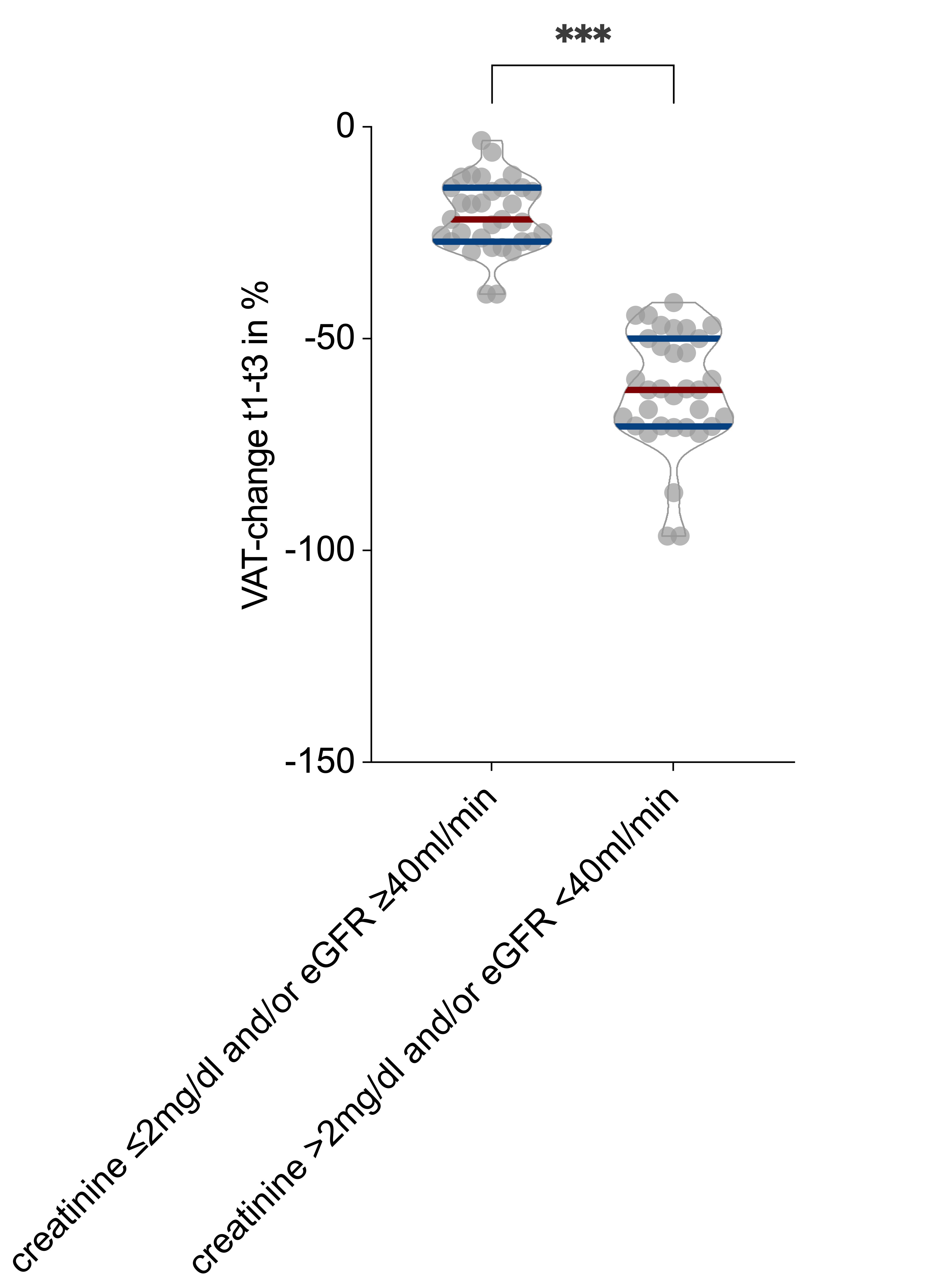

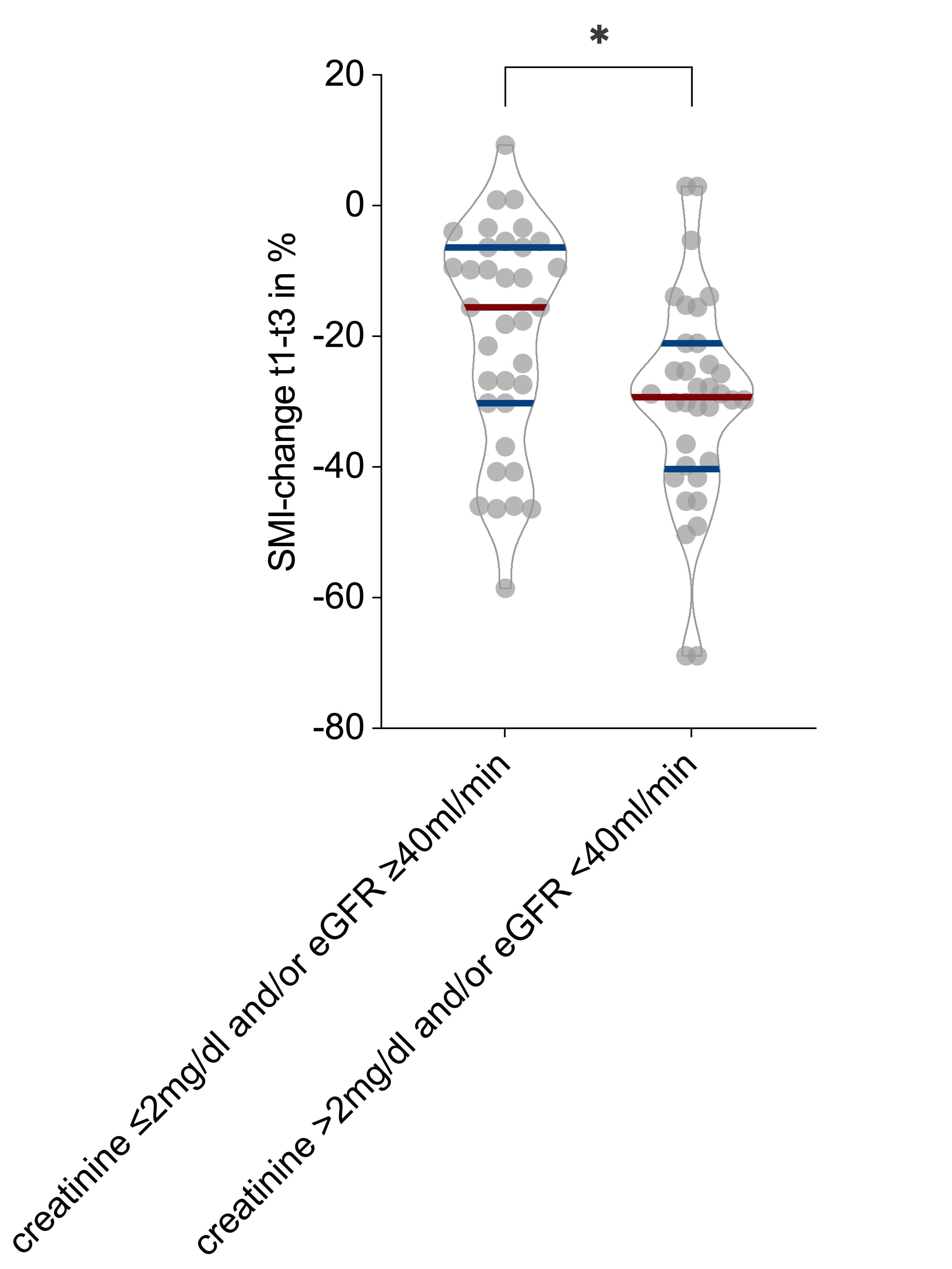

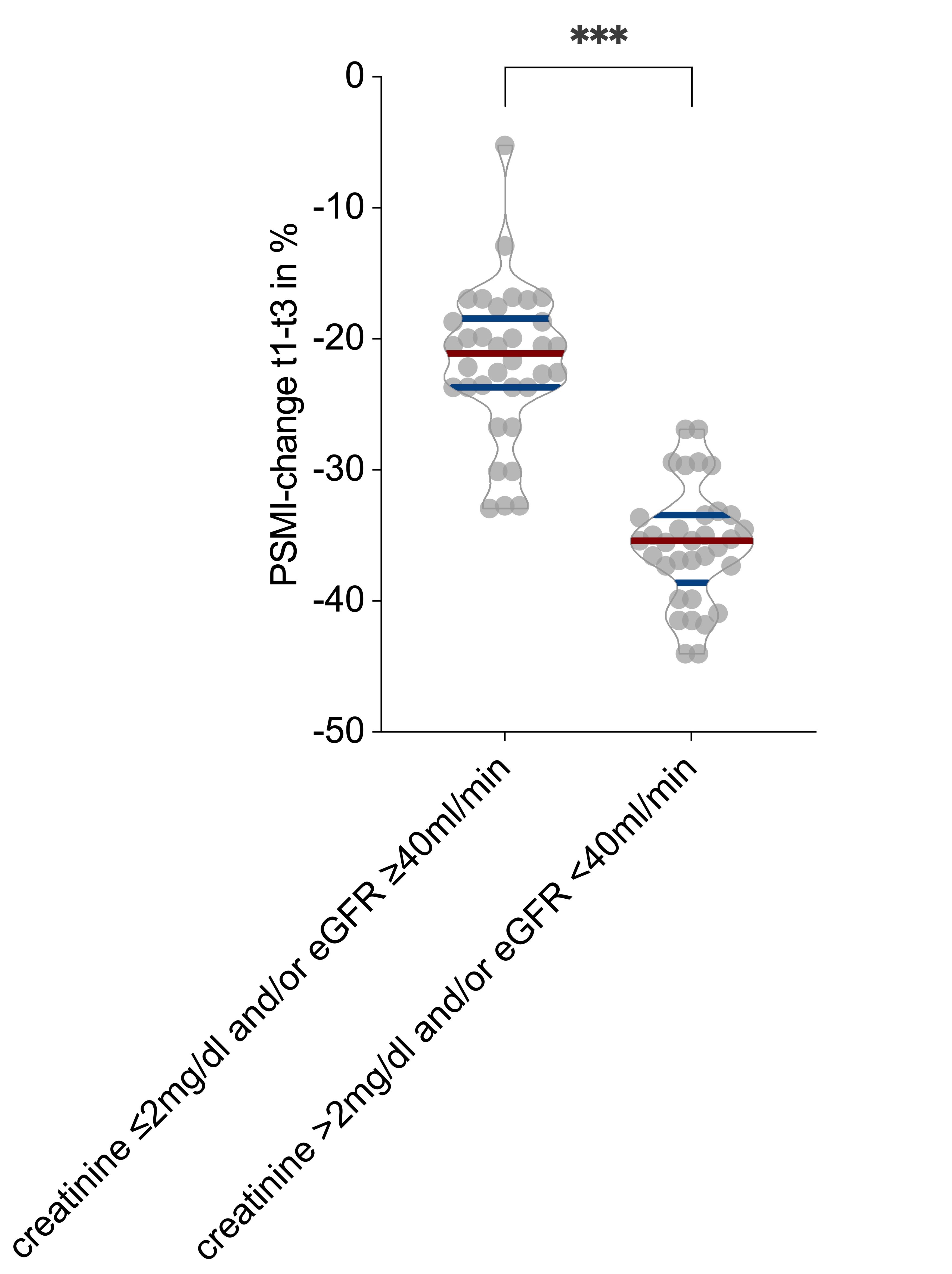

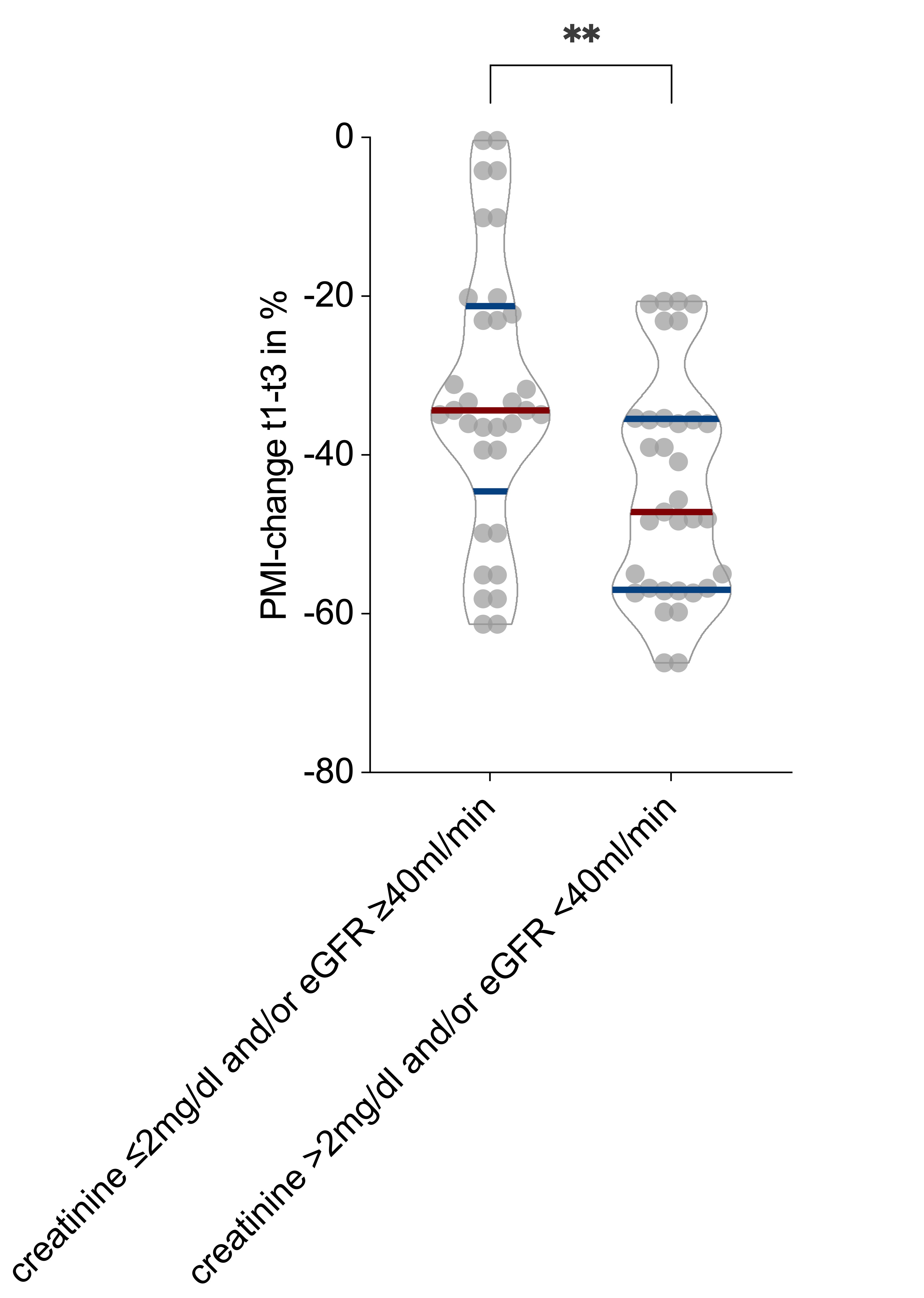


**Supplementary Figure 4 - Percentage change in CT-derived body composition parameters between tCT1 and tCT3 stratified according to IMWG renal impairment criteria.** Shown are changes in (A) skeletal muscle index (SMI), (B) paraspinal muscle index (PSMI), (C) psoas muscle index (PMI), (D) skeletal muscle density (SMD), and (E) visceral adipose tissue (VAT). Patients fulfilling IMWG renal impairment criteria demonstrated greater deterioration across all assessed morphometric parameters.
